# EGF receptor in organ development, tissue homeostasis and regeneration

**DOI:** 10.1186/s12929-025-01119-9

**Published:** 2025-02-19

**Authors:** Claudia Tito, Silvia Masciarelli, Gianni Colotti, Francesco Fazi

**Affiliations:** 1 Department of Anatomical, Histological, Forensic & Orthopaedic Sciences, Section of Histology & Medical Embryology, Sapienza University of Rome, Via A. Scarpa, 14-16, 00161 Rome, Italy; 2 Institute of Molecular Biology and Pathology, Italian National Research Council, IBPM-CNR, C/O Dept. Biochemical Sciences Sapienza University of Rome, Ed. CU027, P.Le A. Moro 5, 00185 Rome, Italy

**Keywords:** EGFR, EGF ligands, Tissue homeostasis, Mammalian development, Signaling

## Abstract

The epidermal growth factor receptor (EGFR) is a protein embedded in the outer membrane of epithelial and mesenchymal cells, bone cells, blood and immune cells, heart cells, glia and stem neural cells. It belongs to the ErbB family, which includes three other related proteins: HER2/ErbB2/c-neu, HER3/ErbB3, and HER4/ErbB4. EGFR binds to seven known signaling molecules, including epidermal growth factor (EGF) and transforming growth factor-alpha (TGF-α). This binding triggers the formation of receptor pairs (dimers), self-phosphorylation of EGFR, and the activation of several signaling pathways within the cell. These pathways influence various cellular processes like proliferation, differentiation, migration, and survival. EGFR plays a critical role in both development and tissue homeostasis, including tissue repair and adult organ regeneration. Altered expression of EGFR is linked to disruption of tissue homeostasis and various diseases, among which cancer. This review focuses on how EGFR contributes to the development of different organs like the placenta, gut, liver, bone, skin, brain, T cell regulation, pancreas, kidneys, mammary glands and lungs along with their associated pathologies. The involvement of EGFR in organ-specific branching morphogenesis process is also discussed. The level of EGFR activity and its impact vary across different organs. Factors as the affinity of its ligands, recycling or degradation processes, and transactivation by other proteins or environmental factors (such as heat stress and smoking) play a role in regulating EGFR activity. Understanding EGFR’s role and regulatory mechanisms holds promise for developing targeted therapeutic strategies.

## Background

Growth factor receptors are transmembrane proteins that activate downstream intracellular signaling cascades upon binding to their specific growth factor ligands. These ligands are peptides, proteins, or hormones that bind to the extracellular domain of growth factor receptors, located on the plasma membrane as monomers. Most growth factor receptors belong to the tyrosine kinase receptor family (RTKs), characterized by tyrosine kinase activity in their intracellular cytoplasmic domain, which is found in a conformation of cis-autoinhibition in the absence of ligand. The ligand binding induces omo- or hetero-dimerization of the receptor, which undergoes a conformational change that triggers its intracellular kinase activity, leading to phosphorylation of downstream proteins involved in the regulation of different cellular processes, including cell proliferation and differentiation, survival, migration, and metabolism [1, 2]. The surface distribution and availability of receptors to bind to ligands are highly regulated processes. Indeed, receptors undergo an endocytosis process which includes either their recycling or degradation, depending on the ligand concentration and the type of receptor [3]. The RTK superfamily comprises 58 receptors, subdivided into multi-member subfamilies such as fibroblast growth factor receptors (FGFRs), insulin and insulin-like growth factor receptors (IR and IGF-1R), platelet-derived growth factor receptors (PDGFRs), vascular endothelial growth factor receptors (VEGFRs) and epidermal growth factor receptors (EGFR/HER/ErbBs) [3]. Expressed in many tissues, these receptors play a vital role in mouse and human physiological development and tissue homeostasis, controlling embryonic development and organogenesis processes. Due to their critical role in regulating various cellular pathways, altered expression or aberrant activation of these receptors can lead to various pathologies and cancers. Several factors contribute to RTK deregulation, including chromosomal translocations, kinase domain duplications, genomic amplifications, gain-of-function mutations, and alterations in endocytic/trafficking processes. One of the most frequently mutated or overexpressed RTK in cancers is the epidermal growth factor receptor (EGFR). EGFR was discovered half a century ago by Stanley Cohen, who—studying the physiological role of the epidermal growth factor (EGF) in murine submaxillary glands—observed its interaction and binding with the specific receptor on plasma membrane of cells [4]. Extensive research has led to the characterization of the structure and signaling of EGFR, identification of its mutations [5] and gene amplification, definition of its role in cancer development [6], and consequently, the development of tyrosine kinase inhibitors as anti-cancer therapies [7]. This review aims to provide a general overview of EGFR’s role in the physiological development of different organs, in tissue homeostasis and in diseases.

## EGFR

### EGFR ligand and signaling pathways activation

**Fig. 1:**
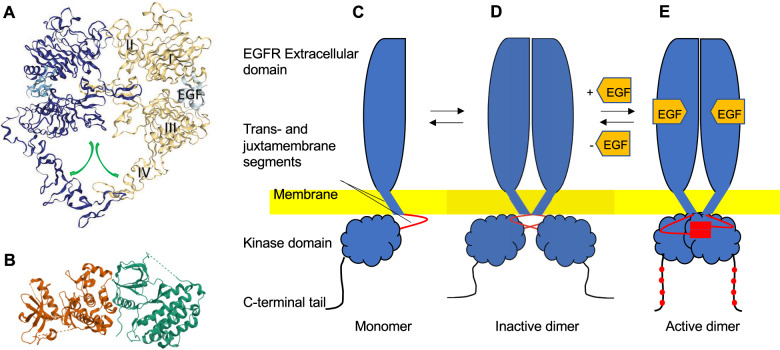
EGFR structure and activation. The EGFR has an extracellular domain, trans- and juxtamembrane segments, and an intracellular kinase domain. **A** Crystal structure of the EGF-bound EGFR ectodomain homodimer (PDB: 8HGS) [252]. The EGFR extracellular domain has four flexible domains (I, II, III and IV). In the absence of ligand, the monomeric extracellular domain assumes a tethered conformation that precludes dimerization. Upon EGF binding (between domains I and III), a conformational change occurs in particular at domain IV (green arrows), resulting in exposure of the dimerization arm of domain II (red circle), thereby increasing dimerization. **B** Crystal structure of the active EGFR kinase domain dimer (PDB: 2GS2) [253]. The domain is intrinsically autoinhibited when monomeric, and is activated by intermolecular interaction, with the formation of an asymmetric dimer. **C**–**E** Cartoons describing EGFR activation. **C** The EGFR monomer is inactive. **D** An inactive dimer may be formed, according to Arkhipov et al. [254]. **E** Activation occurs upon EGF binding, that stabilizes the dimeric form, determining dimerization of the juxtamembrane segments, and formation of asymmetric (active) kinase dimers

**Fig. 2:**
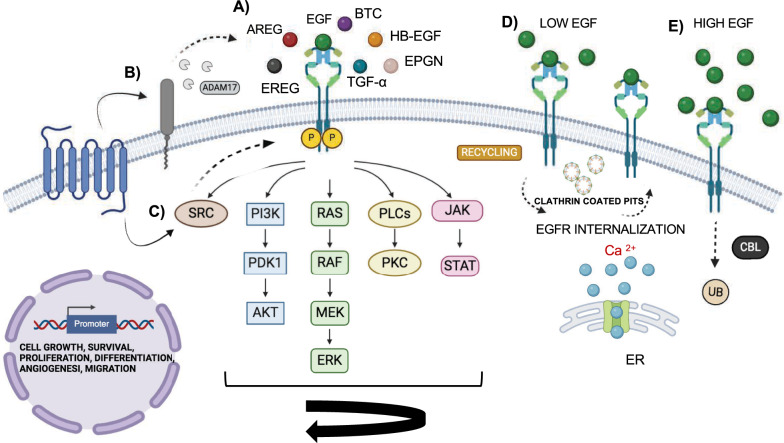
EGFR ligands and signaling pathways activation. **A** EGFR activation: Seven ligands, including EGF, HB-EGF, AREG, TGF-α, epiregulin, EREG, BTC, and EPGN bind EGFR, promoting its dimerization and autophosphorylation which activates downstream signaling pathways, such as ERK MAPK, PI3K-AKT, SRC, PLC-γ1-PKC, JNK and JAK-STAT. These proteins regulate several genes involved in the regulation of different cellular process: cell growth, survival, proliferation, differentiation, angiogenesis and migration. **B**, **C** EGFR trans-activation: G protein-coupled receptor (GPCR)-mediated process leads to EGFR signaling activation by two mechanisms: **B** Activation of metalloprotease group, ADAMs protein that cleave EGFR ligands and release them into the extracellular space. These ligands bind EGFR triggering the signaling cascade activation. **C** Activation of intracellular SRC protein tyrosine kinases involved in the phosphorylation of EGFR in its cytosolic domain, representing a ligand-independent mechanism. **D**, **E** EGFR internalization: Upon EGFR activation, it undergoes to two endocytosis mechanism due to different EGF concentration. Calcium release from ER helps EGFR internalization. **D** At low EGF level, EGFR is incorporated into clathrin-coated pits and is rapidly internalized to endosomes in a complex process named clathrin-mediated endocytosis (CME). Then, EGFR is recycled back to the plasma membrane, leading to signal propagation. **E** At high EGF level, EGFR is internalized, ubiquitinated by E3 ligase Cbl and sorted to lysosomes for protein degradation through a process named non-clathrin-mediated endocytosis (NCE)

The epidermal growth factor receptor (EGFR/HER1/ErbB1) is a member of the ErbB family of RTK receptors, which includes three other proteins, i.e., HER2/ErbB2/c-neu, HER3/ErbB3, and HER4/ErbB4. The structure of EGFR (Fig. 1A, B) consists of three portions: an extracellular region, constituted by four domains, that mediates ligand binding and dimerization, a single hydrophobic transmembrane domain, and an intracellular kinase domain (Fig. 1C–E) [6]. The activation of EGFR may depends on the binding of seven ligands: epidermal growth factor (EGF), heparin-binding EGF-like growth factor (HB-EGF), amphiregulin (AREG), transforming growth factor-alpha (TGF-α), epiregulin (EREG), betacellulin (BTC), and epigen (EPGN) [8]. Upon ligand binding, EGFR undergoes dimerization, autophosphorylation of the tyrosine residues within C-terminal cytoplasmic region and recruitment of adaptor proteins containing phosphotyrosine (pY)–binding Src homology 2 (SH2) domains, through interaction with its phosphorylated tyrosines. These proteins trigger several signal transduction cascades, such as extracellular signal-regulated kinase (ERK-MAPK), PI3K-AKT, SRC, PLC-γ1-PKC, JNK and JAK-STAT pathways that promote the activation of many cellular pathways, including cell proliferation, differentiation, migration, angiogenesis and cell death (Fig. 2A) [9]. Since all the processes are crucial for the correct development of organisms, EGFR activation and signaling pathways regulation results to be essential for physiological development. The tissue homeostasis is maintained through the intracellular balance of apoptosis and cell survival signals determined by ERK/AKT activity pulses depending on canonical EGFR signaling. It represents a finely-regulated spatiotemporal process that promotes a tolerable cell density and tissue integrity [10].

The different EGFR signaling pathways activation depends on the type of ligand and on its binding affinity to the receptor, which can lead to the formation of stable or weak homodimers and of heterodimers with the other ErbB receptors. EGF, TGF-α, HB-EGF, and BTC are high-affinity ligands, whereas AREG, EREG, and EPGN are low-affinity [8]. Recently, bioinformatic tools and analysis of molecular dynamics and crystallographic structure have elucidated how specific ligands promote different effects on EGFR signaling pathways. The binding of EREG and EPGN to EGFR causes a different conformation of the dimer, which is less stable though able to induce a sustained EGFR and ERK activation in time. This kind of ligand binding favors a response leading to cellular differentiation rather than proliferation, which is due to AKT downstream proteins [11]. Moreover, these different cellular processes can be influenced by a single mutation of amino acids in the ligand: the K48T and D46T EGF mutants determine an increase of cellular differentiation compared to wild-type EGF and other EGF mutants that, instead, display an increase of cellular proliferation [12]. Furthermore, comparing the high-affinity ligands, it has been demonstrated that EGF triggers stronger phosphorylation of EGFR and activation of ERK signaling compared to TGF-α, due to the different capability of these ligands to stabilize the Domain IV leg in the tips-separated conformation [13]. Also, considering ERK to be one of the most important EGFR downstream pathways, Lin et al. showed that the re-expression of EGF, TGF-α, and EREG, but not HB-EGF, restored ERK activation in mice knock out for these ligands [14]. Altogether, these data suggest that EGFR ligands are among the most important regulators of EGFR signaling activation, contributing to controlling its activity on tissues homeostasis and organ development. However, EGFR activation can occur also by G protein-coupled receptor (GPCR)-mediated process, called transactivation, which includes two mechanisms. The first involves a matrix metalloprotease (MMP) group, i.e., the ADAM (a disintegrin and metalloproteinase) family. GPCR-induced EGFR transactivation by the activation of MMP enzymes that cleave EGFR ligands and release them into the extracellular space. Thus, the free ligands bind to EGFR which triggers the signaling cascade activation (Fig. 2B). The second one is a ligand-independent mechanism, which implicates the activation of intracellular SRC protein tyrosine kinases by GPCR, and the phosphorylation of EGFR in its cytosolic domain (Fig. 2C). As a result, the phosphorylated EGFR promotes the activation of downstream multiple signaling pathways [15, 16].

### EGFR regulation by endocytosis and ubiquitination processes

The spatiotemporal regulation of EGFR depends on its endocytosis trafficking and lysosomal degradation. Following EGFR activation, its internalization can occur by two different mechanisms depending on EGF concentration: clathrin-mediated endocytosis at low EGF concentration (CME) and non-clathrin-mediated endocytosis at high EGF concentration (NCE). Both mechanisms lead to the internalization of EGFR, which is trafficked to the early endosome compartment; however, while the former leads to EGFR recycling back to the plasma membrane, the latter causes EGFR ubiquitination and degradation via lysosomes. CME is a highly evolutionarily conserved process that occurs through formation of clathrin-coated pits, arising from the invagination of the plasma membrane. It is characterized by different clathrin adaptor and scaffold proteins necessary for the EGFR internalization, such as caveolin, heterotetrameric adaptor protein AP2 complex, epidermal growth factor receptor substrate 15 (EPS15), epidermal growth factor receptor substrate 15-like 1 (EPS15R) and GTPase dynamin (Fig. 2D) [17, 18]. Instead, NCE is a dynamin-dependent process that causes the ubiquitination of EGFR by recruitment of the E3 ligase Cbl, in complex with Grb2, at EGFR phosphorylation sites via direct binding to pY1045 of EGFR and indirect binding to pY1068 and pY1086 sites through GRB2 adaptor. Ubiquitinated EGFR is sorted to lysosomes for protein degradation (Fig. 2E). The Cbl protein exists in three different isoforms, including Cbl, Cbl-b and Cbl-c. All these participate in EGFR ubiquitination but show a different interplay for the distinct phosphorylation sites of EGFR, its endocytic trafficking and signaling activation [19, 20]. Interestingly, Caldieri et al. identified key regulators of NCE process, such as the endoplasmic reticulum (ER)-resident protein reticulon-3 (RTN3) [21]. Knock-down (KD) of RTN3 impairs EGFR internalization at a similar level as dynamin KD, suggesting the involvement of RTN3 in the NCE process. Indeed, they found out that RTN3 is indispensable to promote the site contact between Endoplasmic Reticulum (ER) and plasma membrane (PM) to form the tubulation invagination of the membrane. The release of intracellular calcium from ER, upon activation of the inositol triphosphate (IP3) receptor (IP3R) by EGF-EGFR binding, helps the fixation of tubular invagination and consequent internalization of EGFR [21]. Of note, the role of intracellular calcium release from ER in favoring the internalization of EGFR has been also observed in CME mechanism, suggesting its importance in regulating EGFR recycling and degradation. For instance, in non-small cell lung cancer cell line, the silencing of Sorcin, a calcium-binding protein that increases calcium content in the ER, impairs EGFR signaling and the RAS/ERK signaling cascade at low EGF concentration, affecting cellular migration and invasion [22, 23]. These effects could be linked to the deregulation of calcium in the ER which, affecting the internalization of EGFR, causes an impairment of EGFR downstream signaling pathways. Considerably, EGFR recycling and degradation by CME and NCE mechanisms influence the sustaining of EGFR signaling response. Consequently, the altered regulation of these processes is relevant for cancer development. A manipulation of the balance between EGFR recycling and EGFR degradation could be a novel therapeutic strategy, as demonstrated in the work of Lonic at el. They showed that the phosphorylation of kinase PKCδ stabilizes RAB5-early endosome and inhibits EGFR degradation by preventing the maturation of late endosomes and favoring EGFR recycling back to the plasma membrane. As a result, there is an increase in cellular growth due to prolonged EGFR signaling pathways. On the contrary, the dephosphorylation of pY374-PKCδ by tumor suppressor PTPN14 redirects the endosomal axis towards the degradation pathways [24].

Besides the canonical activation of EGFR through ligand-binding, a non-canonical activation of EGFR occurs by p38-dependent phosphorylation. It is a ligand-independent process triggered by different stimuli, such as inflammatory cytokines as TNF-α, ultraviolet radiation, DNA-damaging agents, and other cellular stress, which leads to rapid internalization of EGFR via CME and consequent recycling of the receptor to plasma membrane [25]. Specifically, this mechanism provides the interaction between the receptor dileucine motif with the σ2 subunit of AP2, enhancing the EGFR endocytic trafficking. Indeed, p38 activity contributes to induce a further increase of the EGFR internalization rate [26]. The amount of EGFR bound to ligand and available on the plasma membrane changes depending on the EGF concentration. At low EGF concentrations, the cell surface has a higher number of unbound EGFRs, which can be activated by other stimuli. For example, under low EGF conditions (≤ 1 ng/ml), high expression of TNF-α due to stress further increases the fraction of internalized EGFRs, by binding to free EGFR. However, p38 non-canonical phosphorylation does not affect the endocytosis of ligand-bound EGFRs. This is likely because receptor kinase activity promotes the recruitment of Grb2, which might sterically hinder the interaction of EGFR with AP2, thereby preventing p38-dependent endocytosis [27]. These findings suggest that EGFR internalization is favored under low EGF conditions compared to high EGF conditions due to both ligand-dependent and ligand-independent activation mechanisms.

## EGFR role in the mammalian development

As described above, EGFR activation initiates a series of downstream signaling pathways, regulating various cellular processes like differentiation, proliferation, survival, apoptosis, blood vessel formation (angiogenesis), and cell movement (migration). Consequently, the alteration of its expression leads to cancer development [28–31]. However, at the physiological level, EGFR is expressed in the placenta and other organs, including lungs, liver, kidneys, intestines, skin, brain, mammary and submaxillary glands, governing pre-implantation and peri-implantation embryo development, gestation period, tissue homeostasis and adult organ regeneration.

Below, we will explore the newly discovered roles of EGFR in the development of different organs. We will analyze its function in regulating tissue homeostasis and regeneration, as well as its contribution to the development of pathologies.

### EGFR in the placenta and embryonic development

**Fig. 3:**
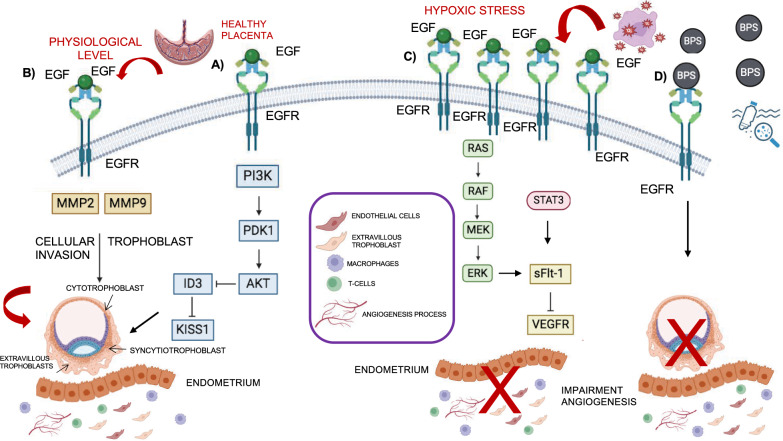
The EGFR role in the placenta development. This image shows both EGFR role in the generation of healthy placenta and in the Preeclampsia disease, due to its dysregulation. **A**, **B** Healthy placenta: **A** EGFR leads to a reduction of KISS1, via AKT activation and ID3 downregulation, promoting trophoblast cell invasion, essential to anchor placental villi to the maternal decidua. **B** Trophoblast cellular invasion and embryo implantation are favored by extracellular matrix (ECM) remodeling due to activation of different metalloproteases expression, such as MMP9 and MMP2, upon EGF/EGFR signaling in trophoblast cells. **C**, **D**, Preeclampsia disorder: **C** The upregulation of EGFR, caused by hypoxic condition, leads to the activation of ERK1/2 and STAT3, and induces the secretion of the antiangiogenic molecule sFlt-1 (soluble FMS-like tyrosine kinase-1) from placenta. sFlt-1 sequesters VEGF and impairs VEGFR activation, affecting angiogenesis process in the placenta. **D** Bisphenol S (BPS) plays a role as ligand of EGFR, impairing its downstream signaling pathways activation and causing a reduction of proliferation and invasion of extravillous trophoblast cells. These events affect the development of placenta

The global analysis of protein expression in human tissues has revealed that it is the placenta where EGFR is more abundant, suggesting a crucial role of the receptor in the regulation of different stages associated to embryonic development [32]. Specifically, EGF and EGFR drive cellular proliferation and placenta differentiation. Specifically, at the 4–6 weeks of gestation age, EGFR and EGF are expressed in the cytotrophoblast cells of the placenta where they stimulate their cellular proliferation. Then, at weeks 6–12, both EGF and EGFR are localized in the differentiated cells of cytotrophoblast, the syncytiotrophoblast cells (involved in the productions of important placental hormones such as human chorionic gonadotropin and human placental lactogen production) and the extravillous trophoblasts (EVTs) [33]. The latter constitute cell columns at the tips of the chorionic villi that acquire migratory and invasive properties, allowing the anchor of placental villi to the maternal decidua [34]. Of note, the alteration of trophoblast invasion represents one of the key factors that cause intrauterine growth restriction and preeclampsia (PE). PE is a gestational hypertension disorder which implicates an impairment of uteroplacental perfusion and, consequently, an impairment of multiorgan development, causing severe complications to fetus and mother [35]. One of the mechanisms through which EGF regulates the health of the placenta is the EGFR-AKT-KISS1 signaling. In vitro experiments*,* carried out using trophoblast cells, showed that EGF treatment affects the expression of KISS1, a placenta-derived hormone, confirming the obtained results by RNA-seq. These data correlated with the high expression of KISS1 in PE patients characterized by insufficient trophoblast cell invasion. Indeed, at the physiological level, EGF stimulates cell invasion through downregulation of ID3 (inhibitor of DNA-binding protein) that mediates the downregulation of KISS1 (Fig. 3A) [36]. Moreover, EGF/EGFR signaling in trophoblast cells activate the expression of different metalloproteases, such as MMP9 and MMP2, which are involved in the extracellular matrix (ECM) remodeling, in order to favor cellular invasion that correlates with trophoblast implantation and the increase of placental perfusion as well as the formation of new blood vessels (angiogenesis) (Fig. 3B) [37, 38]. As in cancer development, the dysregulation of EGFR impairs placenta development, causing PE disorder. Hypoxic conditions upregulate EGFR expression which, activating downstream pathways (ERK1/2 and STAT3) that induce the secretion of the antiangiogenic soluble FMS-like tyrosine kinase-1 (sFlt-1) from the placenta. sFlt-1 sequesters VEGF and causes a reduction of VEGFR activation which impairs vascular homeostasis and endothelial dysfunction (Fig. 3C). Beyond EGFR activation, mitochondrial function also increases sFlt-1 secretion from primary cytotrophoblasts via activation of energy-sensing molecules, like AMPK, SIRT1, PGC1α. However, these two pathways are independent. EGFR inhibitor (EGFRI) as Gefitinib and energy-sensing molecules activators (resveratrol) reduce circulating sFlt-1 levels in pregnant mice, suggesting possibly relevant therapeutic targets for PE [39, 40]. Bisphenol S (BPS), endocrine-disrupting chemical which can be found in the plastic containers for food and drink, competes with EGF for EGFR binding, interfering with EGFR internalization and recycling [41, 42]. This disrupts EGFR's functional effects, leading to reduced proliferation, invasion, and tube formation in extravillous trophoblast cells (Fig. 3D) [43]. EGFR knockout (KO) mice exhibit defects in the decidualization, a process that prepares the endometrium for embryo implantation, highlighting the crucial role of EGFR in placental health and progression of early pregnancy [44]. Depending on genetic background, KO mice can have different fates: they can die at early or mid-gestation, likely of dysregulation of placenta formation, or they can survive at birth until postnatal 8–20 days. In this latter case, they are characterized by different defects in different organs development, like skin, brain, lung and gastrointestinal tract [45, 46].

Based on these findings, EGFR represents a key factor for placental health and a promising molecular drug target for placental diseases. Recently, Geisler et al. have developed ionizable lipid nanoparticles (LNPs) conjugated with EGFR antibodies, able to increase EGFR uptake in placental trophoblasts, suggesting a potential therapeutic strategy for improving pregnancy outcome [47].

### Gut development

The intestinal epithelium, a dynamic layer of the digestive system, is composed of different cell types, including enterocytes, goblet, enteroendocrine, and Paneth cells. It plays a pivotal role in digestion, nutrition absorption and defense against microorganisms, allergens, antigens, and toxins [48, 49], by forming a selective barrier against harmful substances, which separates the intestinal mucosa from the lumen environment [50]. Intestinal epithelial cells (IECs) are responsible for tissue homeostasis, regulating the intestinal barrier via a high rate of cell turnover, which includes cellular differentiation and proliferation processes. Tight junction, adherens junctions and desmosomes maintain the integrity of the barrier, and their deregulation leads to the increase of gut permeability and is associated to various diseases [51]. Several signaling pathways, including EGF, Notch and WNT, regulate IEC proliferation and intestinal stem cells (ISCs) proliferation, while Bone Morphogenetic Protein (BMP) controls differentiation, counteracting stemness maintenance [52]. The involvement of the EGF-EGFR pathway in intestinal homeostasis, nutrient absorption and tissue regeneration has been investigated in humans, mice and even Drosophila.

**Fig. 4:**
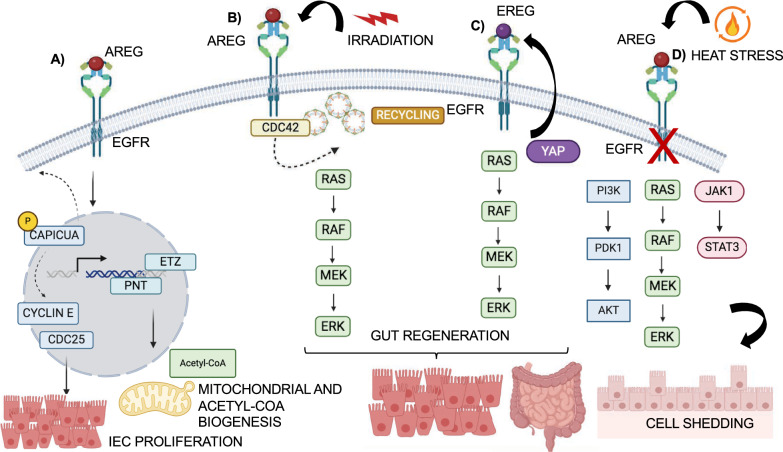
The EGFR roles in IECs proliferation and gut regeneration. This image highlights the various ways EGFR contributes to IEC proliferation and gut regeneration. Here's a breakdown of the mechanisms: **A** EGF-EGFR Binding: High levels of EGFR and its ligand EGF are found in epithelial intestinal cells. EGF binding to EGFR triggers proliferation both directly and indirectly. Directly, EGFR signaling activation induces phosphorylation of the nuclear repressor factor Capicua, promoting its translocation into the cytoplasm, and the consequent transcription of cell cycle regulators, such as Cdc25 and Cyclin E. Indirectly, EGFR leads to the activation of Pnt and ETZ transcription factors involved in the mitochondrial biogenesis pathways. **B** EGFR Internalization and Activation: Following gut injury (e.g., from radiation), a protein called Cdc42 interacts with clathrin in the endocytosis complex, favoring EGFR internalization and activation of downstream signaling pathways, that ultimately lead to cellular proliferation and tissue regeneration. **C** YAP and EGFR Signaling: YAP is another interactor of EGFR. YAP induces EGFR signaling by promoting the production of a growth factor called EREG in the surrounding tissue (stroma). EREG can then bind to EGFR, further controlling the balance between proliferating IECs and the differentiation of these cells into mature intestinal epithelial cells. **D** Heat Stress and EGFR: Heat stress disrupts EGFR signaling pathways, leading to the shedding of cells and ultimately to a breakdown of the intestinal barrier

EGFR and its ligand EGF are found in epithelial cells and adjacent fibroblasts within the crypts, acting as key players in stem cell proliferation by activating MAPK and AKT signaling pathways [53]. The MAPK downstream pathway is particularly important for EGF-mediated effects. Studies in Drosophila, a model system with a gut structure similar to mammals, have shown that EGFR/MAPK activation stimulates cellular growth and proliferation in two ways: (1) indirectly: phosphorylating a repressor factor (Capicua), thereby causing its translocation from the nucleus to the cytoplasm, and leading to the transcription of cell cycle regulators, such as Cdc25 and Cyclin E; (2) directly: activating Pnt and ETZ transcription factors [54]. A bioinformatic analysis showed that many genes activated by Pnt and ETZ are primarily involved in the mitochondrial biogenesis pathways, oxidative phosphorylation (OXPHOS), mitochondrial ATP biogenesis, acetyl-CoA biogenesis, and oxidation–reduction. All these processes are crucial for cellular proliferation. Accordingly, the downregulation of EGFR in mammalian IECs reduces the expression of mitochondrial transcription factor A (TFAM) and dimethyladenosine transferase 2, mitochondrial (mtTFB2), essential for mitochondrial biogenesis and activity and IEC proliferation. Moreover, EGFR can enhance cell metabolism, glucose uptake, glycolysis, and synthesis of amino acids and nucleotides, contributing to cellular proliferation (Fig. 4A) [55].

Other studies have investigated EGFR’s role in IECs proliferation during regeneration after injury. An interesting regulatory mechanism involves the cell division control 42 protein (Cdc42), a Rho small GTPase, involved in multiple actin-dependent processes, such as cytoskeletal reorganization and cell polarity. Cdc42 expression in IECs promotes epithelial tissues regeneration, while its loss reduces crypts regeneration, leading to inflammation and increased injury susceptibility [56]. This susceptibility might be linked to EGFR expression. Cdc42, especially isoform 2, interacts with clathrin proteins in the endocytosis complex, facilitating EGFR internalization and activation of survival pathways. This promotes increased cellular proliferation and tissue regeneration, upon injury by irradiation (Fig. 4B) [57]. However, other pathways like mTOR and Yap-Hippo also play vital roles in IEC proliferation during regeneration [58, 59]. The Yap-Hippo pathway promotes cell survival and regeneration by inducing EGFR signaling through stromal EREG expression, independent of EGF. Notably, in Yap-deficient organoids the morphogenesis of crypts is altered, and the ectopic expression of the EGFR ligand EREG overcomes the injury by induction of a regenerative signaling [59]. The YAP-EGFR axis regulates the balance between IECs and proliferating transit amplifying (TA) cells that differentiate into mature intestinal epithelial cells during homeostasis (Fig. 4C) [60]. Thus, the YAP protein could represent a potential target to disrupt the EGFR signaling cascade and influence cellular survival and proliferation.

The balance between sustained and attenuated EGFR signaling depends on recycling back to the plasma membrane or lysosomal degradation. Alterations in genes involved in these processes affect EGFR's impact on IEC proliferation through recycling [61]. Conversely, the Notch pathway antagonizes EGFR-dependent proliferation by activating programmed cell death through regulation of pro-apoptotic genes, leading to downregulated EGFR expression [62]. Maintaining the balance between cell death and survival in IECs is crucial for intestinal homeostasis. Understanding the genes involved in these processes could be valuable in developing strategies to prevent uncontrolled IEC renewal and tumorigenesis.

The integrity of the intestinal barrier is essential for maintaining tissue homeostasis. IECs form a selective barrier with tight cell–cell communication, and their ability to do so is partly determined by EGFR expression. Through a MAPK-dependent pathway, EGFR suppresses intestinal epithelial cell shedding [63]. Heat stress triggers shedding, which weakens the intestinal barrier and disrupts junctional complexes [64]. One mechanism by which heat stress might influence gut homeostasis is through the impairment of EGFR signaling. Wang et al. demonstrated in vitro that EGF induces STAT3, AKT, and ERK1/2 in a dose- and time-dependent manner in control samples, but not in those under heat stress conditions (Fig. 4D). This suggests a significant inhibition of EGF/EGFR intracellular signaling under heat stress [65]. Moreover, the expression of EGFR in non-epithelial intestinal cells, such as myofibroblasts, and in specific intestinal stromal cells plays an important role in chronic inflammatory conditions, such as the inflammatory bowel diseases, IBD, including Crohn's disease and ulcerative colitis, which are associated with colorectal cancer (CRC) development [66]. All these diseases are characterized by alteration of immune system and different expression of cytokines and chemokines. Indeed, many CRC patients showed an upregulation of EGFR in myeloid but not in IECs cells that correlated with patient overall survival. Experiment in *vivo* confirmed that EGFR signaling in myeloid cells represents the main player of colitis-associated colorectal cancer (CAC), by activation of STAT3 and survivin genes in intestinal tumor cells. Indeed, in CAC mouse model the deletion of EGFR in myeloid cells reduced significantly the tumor size. Interestingly, EGFR-STAT3 activation in myeloid cells protects mice from colitis induction by dextran sodium sulfate treatment (DDS), via regulation of IL-6 production, which prevents intestinal damage leading to IEC proliferation [67]. Besides to myeloid cells, intestinal macrophages, which are involved in the intestinal homeostasis, control intestinal inflammation by the regulation of cytokines production. Deletion of EGFR in macrophages in DSS-colitis mice leads to the increase of anti-inflammatory cytokines, such as IL-10 that inhibits the release of proinflammatory cytokines such as IL-6, IL-8 and TNF, limiting colitis development [68]. These works demonstrated a complex role for EGFR that might be explained by differences in concentration and duration of DSS treatment and different type of cells.

Taken together, these findings highlight the role of EGFR in IECs proliferation and survival, which are essential for maintaining gut epithelial homeostasis. EGFR achieves this by inhibiting cell shedding and activating pathways involved in cell migration, biogenesis, and glucose metabolism. Additionally, various mechanisms regulate EGFR function, including the activation of genes controlling its endocytosis and recycling to the cell surface, as well as the secretion and release of ligand growth factors.

### Liver development

The material absorbed by the small intestine is processed in the liver. The liver is the body’s largest organ and performs numerous vital functions, such as producing bile, which is necessary for the digestive process; removing waste products and defending against external pathogens and infection; regulating glucose metabolism by controlling blood sugar levels; and producing amino acids. It also possesses a remarkable ability to regenerate after injury or infection, a process carried out by adult quiescent hepatocytes that can acquire proliferation properties [69]. The regeneration process involves three key events. (1) Expression of Cytokine Signaling, like TNF-α and IL-6, that activate transcription factors (AP-1, STAT3, NF-κB), triggering DNA synthesis [70, 71]. In this phase, hepatocytes lose their quiescent state and enter into the mitotic cycle. (2) Activation of Growth Factor like HGF, TGF-α, EGF, HB-EGF, and AREG that act as mitogens and bind their respective receptors, including EGFR and MET, stimulating hepatocyte proliferation and entry into G1 phase of cell cycle. (3) Terminal stage that includes an inhibition of hepatocytes proliferation and maintenance of hepatostat, likely to be caused by TGFβ expression [72–75].

**Fig. 5:**
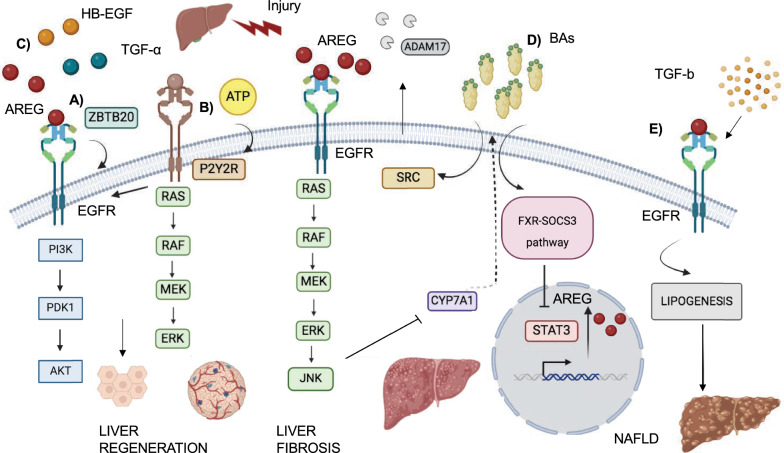
The EGFR roles in liver regeneration and fibrosis. This figure illustrates the multifaceted role of the epidermal growth factor receptor (EGFR) in both liver regeneration after injury and the development of liver fibrosis. **A**, **B**, **C**: EGFR Activation Promotes Liver Regeneration. **A** The ZBTB20 gene promotes the activation of EGFR and AKT signalling pathways, contributing to liver regeneration. **B** P2Y2R, a G protein-coupled receptor, activates EGFR upon binding to extracellular ATP. This activation increases phosphorylated ERK (p-ERK) levels and promotes cellular proliferation. **C** During liver regeneration, various ligands such as HB-EGF, TGF-α, AREG, and epiregulin become elevated. These ligands bind to EGFR, leading to the activation of downstream signalling pathways that promote cell proliferation and ultimately liver regeneration. **D**, **E**: EGFR and Liver Fibrosis. **D** Accumulation of bile acids (BAs) triggers EGFR transactivation, which in turn activates the MAPK/ERK and JNK signalling pathways. These pathways are involved in repressing Cyp7a1, a gene that regulates BA synthesis. Additionally, BAs activate the FXR-SOCS3 pathway, leading to STAT3 inhibition. This allows for the transcription of AREG and other genes by nuclear EGFR. **E** EGFR activation can induce the expression of genes involved in lipogenesis, a process that contributes to non-alcoholic fatty liver disease (NAFLD). NAFLD can progress to non-alcoholic steatohepatitis (NASH), fibrosis, cirrhosis, and even hepatocellular carcinoma

EGFR, expressed in mature hepatocyte cells, plays a crucial role in their proliferation and liver regeneration. It activates signaling pathways like ERK1/ERK2, PI3K/AKT, and PLCγ, promoting the transcription of genes involved in the cell cycle and survival. Interestingly, EGFR is not essential for embryonic liver development but becomes critical for regeneration in adults [76–79]. To this purpose, studies in EGFR-deficient mice showed impaired liver regeneration due to decrease of D1 cyclin and cell cycle arrest after partial hepatectomy (PH). These effects are followed by a compensatory mechanism that comprises TNFα upregulation and c-Jun activation, which reduces the expression of p38α, preventing hepatocytes apoptosis [80]. These data suggest that EGFR primarily drives liver regeneration through its proliferative effects, rather than cellular survival regulation, upon hepatic damage. The study of Lopez-Luque et al., confirms this hypothesis, demonstrating no alteration of apoptosis between wild type and EGFR^−^/^−^ mice. In this model, upregulation of TGFβ induces the expression of p15INK4, an inhibitor of cell cycle progression, leading to delayed liver regeneration after two-thirds PH and activation of the HGF/Met pathway as potential compensatory mechanism. Furthermore, expression of the truncated form of EGFR leads to a delay, but not a prevention, of cytokines expression and HCC development [81]. Altogether, these studies confirmed the essential role of EGFR in the early phases of liver regeneration, regulating the initial hepatocyte proliferation. EGFR regulation in regeneration after injury occurs at different levels. At the cellular level, one of the EGFR signaling regulator is the ZBTB20 gene, a transcriptional repressor of alpha-fetoprotein gene which is a serum marker of hepatocarcinoma [82]. Deletion of ZBTB20 in hepatocytes after partial hepatectomy leads to reduced EGFR expression and AKT signaling, hindering the mitogenic response of hepatocytes to growth factors (Fig. 5A) [83]. Another positive regulator of EGFR is the P2Y2 receptor (P2Y2R), a member of the G protein-coupled P2Y purinergic receptors, which activates EGFR through transactivation. This process involves extracellular ATP binding to P2Y2R, which increases p-ERK phosphorylation and promotes cellular proliferation. However, P2Y2R also plays an oncogenic role, and its pharmacological inhibition can have dual benefits: reducing inflammation after injury and suppressing tumorigenesis (Fig. 5B) [84, 85]. Beyond cellular components, extracellular stimuli as treatment with Genistein, a natural compound of isoflavones, can also transactivate EGFR. Genistein strongly activates the EGFR downstream signaling pathways, in particular AKT and STAT, leading to cellular proliferation and activation of survival pathways linked to liver regeneration [86]. Furthermore, EGFR ligands like HB-EGF, TGF-α, AREG, and epiregulin are elevated during regeneration (Fig. 5C). ADAM 10 and ADAM17 proteases facilitate the release of these ligands. In models lacking these proteins, impaired ligand release leads to reduced EGFR expression and AKT phosphorylation [87].

Cholestatic liver diseases depend on impaired bile flow and/or biosynthesis due to altered hepatobiliary bile acids (BAs) transport, causing bile acid accumulation, and consequent liver damage and fibrosis, that may lead to hepatocellular carcinoma (HCC). Liver fibrosis represents an alteration of tissue homeostasis modulated by the interplay between liver mesenchymal cells and macrophages that are involved in the accumulation of ECM and increase of cytokines and chemokines [88–91]. The role of EGFR in these diseases remains to be better clarified. On the one hand, some studies have demonstrated the protective role of EGFR in cholestatic liver injury and fibrosis that involves STAT3 pathways [92] and the release of EGF ligands, such as AREG. Of note, the high expression of AREG upon cholestasis injury liver, caused by intrahepatic accumulation of BAs that promotes SRC and ADAM17 activation, induces activation of EGFR. EGFR in turns activates MAPK/ERK and JNK signaling pathways involved in Cyp7a1 repression, a gene that regulates BAs synthesis. At the same time, BAs inhibits STAT3 by FXR-SOCS3 pathway activation, allowing the transcription of AREG and other genes by nuclear EGFR (Fig. 5D) [93]. Accordingly, genetically modified mouse models represent relevant systems to better characterize the role of EGFR in liver fibrosis and in the crosstalk between hepatocytes and macrophages cells that influences HCC development. Indeed, the deletion of EGFR in parenchymal cells of mice affected by HCC leads to hepatic death and an increase of IL-1β expression. The latter induces EGFR expression and transactivation in non-parenchymal cells, such as Kupffer cells/liver macrophages, that leads to IL-6 production, stimulating hepatocytes proliferation and increasing HCC formation. The aforementioned study further confirms the role of EGFR in the regulation of inflammatory signals by parenchymal and non- parenchymal cells. Indeed, the truncated form of EGFR allows EGFR ligands to bind to the receptors in the hepatocyte cells and to avoid that the excess of other EGFR ligands remains available to the nonparenchymal cells. The lack of EGFR activation in nonparenchymal cells could lead to delay of the cytokines expression, inflammatory process and tumor lesion development observed in these murine models [81].

Thus, the mechanism of crosstalk between different type of cells upon pathological condition, besides highlighting the role of EGFR in the regulation of inflammatory signals by parenchymal and/or non-parenchymal cells, could be relevant for the therapeutic strategies based on EGFRIs in specific cellular lines [94].

Conversely, other studies suggest an opposite EGFR function in cholestasis liver injury, demonstrating that the deletion of the catalytic domain of EGFR can be beneficial, reducing collagen gene expression, promoting anti-inflammatory responses, and increasing M2 macrophages that suppress fibrosis. However, compensatory mechanisms maintain cellular proliferation through AKT and ERK activation in this scenario [95].

One of the main functions of the liver is to regulate the synthesis and distribution of lipids to tissues, lipid uptake from the circulatory system, and elimination of toxic lipids to maintain tissue homeostasis. The imbalance between these processes leads to fat accumulation and the development of non-alcoholic fatty liver disease (NAFLD), characterized by non-alcoholic steatohepatitis (NASH), fibrosis, cirrhosis, and hepatocellular carcinoma [96, 97]. EGFR levels decrease with NAFLD progression, while its overexpression reduces the expression of genes involved in lipogenesis, such as SREBF1, FASN, ACC1 and PPARα through TGF-β activation pathway. This suggests a potential protective role for EGFR in regulating fat metabolism within the liver (Fig. 5E) [98].

EGFR plays a multifaceted role in liver health and its altered expression is linked to various liver diseases. EGFR is a main actor in liver regeneration, activating protective strategies, such as cellular proliferation and survival of hepatocytes to re-establish the homeostasis of the organ; nevertheless, EGFR activation can be linked to liver fibrogenesis and tumorigenesis, due to prolonged proliferation and regulation of genes involved in the inflammatory and fibrosis process. Identifying regulators of EGFR activity is crucial for understanding its impact on liver tissues and developing novel therapeutic strategies for liver diseases.

### Bone development

Bone development is a regulated process that occurs during embryogenesis, continues until early adulthood and involves two mechanisms: intramembranous and endochondral ossification. Intramembranous ossification begins in utero and includes the differentiation of mesenchymal cells into specialized cells, such as osteoblasts, which form primary ossification centers. They promote the synthesis of extracellular matrix (ECM) components involved in matrix hardening in which osteoblasts are sequestered and transformed in osteocytes. Endochondral ossification consists of the differentiation of mesenchymal cells in chondrocytes that start proliferating and secreting an extracellular matrix in order to form a hyaline cartilage model of the bone. Then, chondrocytes become hypertrophic, contributing to the growth of bone in length and promoting the calcification of the ECM by secretion of collagen X and fibronectin. This calcification prevents the nutrition of chondrocytes, which undergo cell death. Then, the vascularization process carries osteogenic cells, such as osteoblasts and osteoclasts which replace hypertrophic cartilage ECM with bone [99]. As a result, the skeletal development results in a cross-talk between chondrocytes and osteoblasts at various stages of differentiation.

**Fig. 6:**
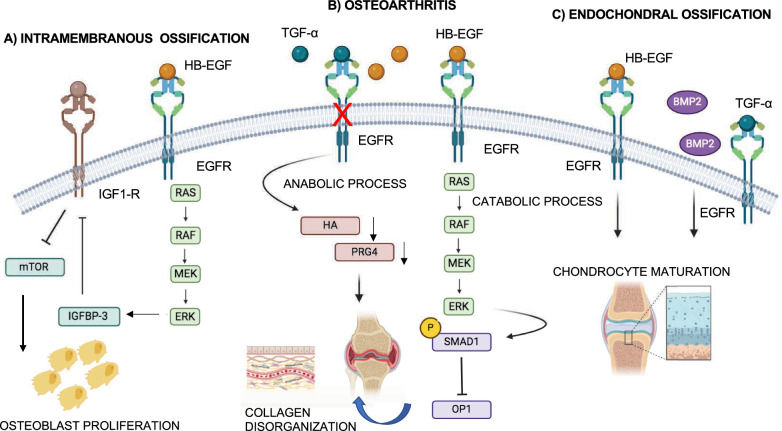
The EGFR roles in bone development. **A** Intramembranous ossification: EGFR activation induces ERK phosphorylation, which increases IGFBP-3 expression. IGFBP-3 represses the IGF-1R signalling involved in the regulation of the mTOR pathways, leading to osteoblast proliferation. **B** Osteoarthritis: EGFR promotes anabolic and catabolic processes. EGFR loss impairs the synthesis of lubricants Prg4 and hyaluronic acid (HA), resulting in a disorganization of collagen fibers. Upon HB-EGF-EGFR binding, ERK phosphorylation and p38 MAP kinases activation leads to phosphorylation of Smad1 and inhibition of OP-1, affecting the synthesis of matrix proteins and favoring osteoarthritis. **C** Endochondral ossification: A crosstalk between EGFR and BMP signals regulates chondrocyte maturation, promoting a correct long bone growth and bone development

EGFR plays a crucial role in both processes of bone development. In intramembranous ossification, EGFR regulates osteoblast maturation (the process by which immature osteoblasts become mature bone-forming cells) and ossification in vivo. Studies in mice lacking EGFR in osteoprogenitors (precursor cells) demonstrate osteopenia (reduced bone density) and bone defects. Mechanistically, EGFR activation inhibits the mTOR pathway, which normally drives osteoblast differentiation. During embryonic and early postnatal development, by inhibiting mTOR pathways, EGFR maintains an appropriate osteoblast proliferation and prevents a premature differentiation of osteoprogenitor cells and mineralization process, leading to proper bone development. Indeed, the activation of EGFR induces ERK phosphorylation, which mediates IGFBP-3 upregulation. IGFBP-3 negatively regulates IGF-1R signaling, leading to repression of mTOR pathways, which drives the osteoblast differentiation from pro-osteoblasts and the synthesis and mineralization of the ECM (Fig. 6A) [100]. EGFR^−/−^ embryos showed upregulation of the chondrocyte transcription factor Runx2, that controls the osteoblast differentiation by transcription of osteoblast-specific genes, and downregulation of Osteocalcin, a non-collagenous protein expressed in osteoblast, involved in the synthesis of ECM. This results in an augmented zone of hypertrophic chondrocytes and mineralization impairment. Accordingly, the inhibition of mTOR in EGFR^−/−^ embryos rescues the proportion between these genes [101].

In endochondral ossification, EGFR and its ligands TGF-α, HB-EGF, amphiregulin and epiregulin are highly expressed in articular cartilage, where they regulate the balance between cartilage building (anabolic) and breakdown (catabolic) processes, playing an essential function in this process. An imbalance favoring breakdown leads to osteoarthritis (OA), a common joint disease characterized by cartilage degeneration [102–104].

EGFR's role in OA is complex. Some studies suggest it promotes anabolic processes, while others suggest it might contribute to catabolism. EGFR deletion in mice initiates and accelerates OA by reducing chondrocyte number, survival, and function. EGFR deletion also affects the synthesis of lubricants Prg4 and hyaluronic acid, resulting in a disorganization of collagen fibers. This suggests therapeutic potential for EGFR ligands like HB-EGF and TGF-α to promote cartilage regeneration in OA treatment [105, 106]. The use of nanoparticles containing TGF-α has been tested in mice after loading injury, demonstrating an attenuation of the disease [107].

However, other studies suggest a role of EGFR in promoting catabolic gene expression rather than anabolic activity. High levels of HB-EGF, an EGFR ligand, can activate pathways leading to cartilage breakdown. Activation of ERK phosphorylation and p38 MAP kinases, upon EGFR binding, leads to phosphorylation of Smad1, associated to inhibition of OP-1, a protein involved in the synthesis of matrix proteins necessary to promote assembly and integrity of ECM. This highlights the need for further research to fully understand EGFR’s role in OA (Fig. 6B) [108–110].

Intriguingly, EGFR also interacts with BMP signaling pathways in the hypertrophic zone of cartilage, a crucial area regulating bone growth, which modulates the balance between the proliferation and maturation of chondrocytes, impacting the growth and length of bone. This dynamic interaction highlights the complex interplay between EGFR and other signaling pathways during bone development. Experiments in vivo using knockout mice for EGFR or BMP have explained this reciprocal regulation in the hypertrophic zone. The loss of EGFR in hypertrophic chondrocytes leads to the increase of pSMAD1/5/8 and collagen X deposition, accelerating chondrocyte differentiation. On the contrary, the loss of endogenous BMP2 activity increases EGFR signaling in the hypertrophic zone, showing shortened hypertrophic zones with altered collagen expression (Fig. 6C) [111]. Thus, the cross-talk between EGFR and BMP signals, based on reciprocal repression, is necessary to promote appropriate chondrocyte maturation, which is important for long bone growth and tissue homeostasis in adults.

EGFR therefore plays a vital role in bone development, regulating osteoblast maturation in intramembranous ossification and influencing the balance between cartilage building and breakdown in endochondral ossification. Further research is needed to fully understand EGFR's complex roles in various aspects of bone biology and its potential as a therapeutic target for OA.

### Skin development

**Fig. 7:**
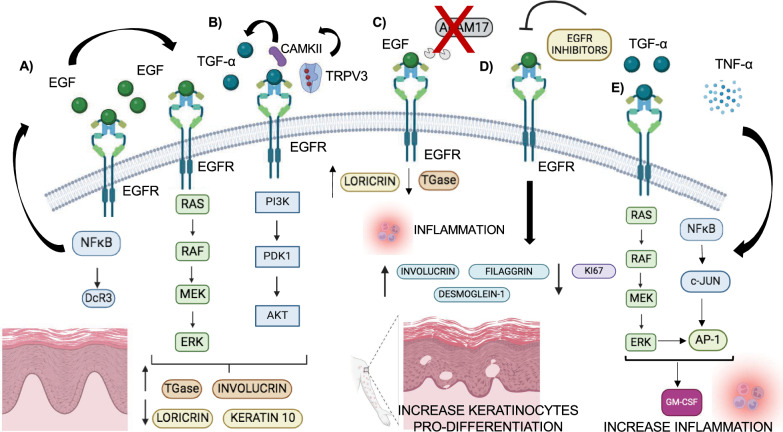
The EGFR role in skin. EGFR protein is prominently expressed in the lower layers of epidermis, whereas it is downregulated during the cornification process. Altered EGFR expression promotes changes in the expression of keratinocytes markers which participate in the cellular proliferation and differentiation. **A**, **B** Upregulation of EGFR: **A** The increased EGFR level leads to upregulation of DcR3 that induces elevated expression of CE markers, such as involucrin and TGase, and reduced expression of early differentiation markers, including keratin 10 and loricrin. As a result, keratinocytes showed an enhanced proliferation. **B** A similar effect is also directed by TRPV3, which plays a role in increasing of calcium influx, promoting the CAMKII, involved in the TGFα release and EGFR activation. **C**, **D** Downregulation of EGFR and side-effects on cancer patients: **C** The lack of ADAMs in mice leads to inactivation of EGFR and immature production of stratum corneum, due to an increase of loricrin levels and a decrease of TGM expression. Consequently, an accumulation of inflammatory and T cell population has been reported, affecting the homeostasis of epidermis and skin inflammation development. **D** Use of EGFRIs in cancer patients leads to side effects such as cutaneous complications. The downregulation of EGFR impairs cellular proliferation in the basal epidermis layers as demonstrated by reduction of Ki67 and leads to pro-differentiation process, upregulating filaggrin, involucrin and Desmoglein-1. As a result, the skin barrier is affected. **E** EGFR and inflammation process: TGF-α leads to EGFR activation and strengthen TNF-α effect, leading to high GM- CSF expression through regulation of activator protein (AP)−1 and NFkB expression. Moreover, TNF-α promotes the phosphorylation of c-Jun involved in the transactivation of AP-1. These events lead to the increase of cytokines and macrophages that are involved in chronic inflammatory disease progression

The epidermidis is a stratified squamous epithelium constituted by five sublayers that act as a protective barrier against pathogens, bacteria, germs and other external mechanical and environmental factors. It is composed by different cell types, including keratinocytes, melanocytes, Langerhans cells and Merkel cells. Keratinocytes, the prevalent cell type, undergo proliferation and differentiation to maintain epidermal morphogenesis and homeostasis. Keratinocytes undergo cellular proliferation in the basal layers, later entering the upper layers where they play a stratification and differentiation process through cellular signaling pathways, cytokines, growth factors and adherens junction expression [112]. The cornification process, characterized by the formation of the cornified envelope (CE), occurs during the final stage of epidermis development. CE is rich in transglutaminase (TGase), that crosslinks with other differentiation marker proteins, such as involucrin, keratin 1/10, profilagrin and loricrin, replacing plasma membrane and constituting an enhanced barrier [113]. EGFR is prominently expressed in the lower layers of epidermis but is downregulated during the cornification process. Here, upregulation of EGFR is associated with psoriasis, via regulation and interaction with other factors, as Decoy receptor 3 (DcR3). DcR3, a soluble receptor of Fas ligand involved in immune response, is upregulated by EGF and TNF- α treatment of epidermis keratinocytes through EGFR activation and NF-κB signaling [114]. Downregulation of DcR3 reduces involucrin and TGase expression, that are crucial for the formation of CE, while increasing keratin 10 and loricrin, expressed in the early differentiation markers, suggesting a role in the regulation of CE formation (Fig. 7A) [115]. TRPV3, a Ca^2+^-permeable transient cation channel, is another factor involved in EGFR activation and keratinocyte proliferation. Chemical activation of TRPV3 promotes calcium influx, activating Calcium/Calmodulin Dependent Protein Kinase II (CAMKII) and leading to the release of TGFα, which stimulates EGFRs in an autocrine manner, causing keratinocyte proliferation by PI3K and NF-κB activation (Fig. 7B) [116]. The EGF/EGFR axis, with its downstream signaling cascade of Src/PI3K/Akt/mTOR, also promotes the motility and invasion ability of oral keratinocytes [117]. Inhibition of the EGF/EGFR axis is correlated with the skin inflammation and disorders, altering the epidermis homeostasis, reducing the protective barrier, and increasing susceptibility to bacterial infections. Mice lacking EGFR or ADAM17 expression show similar phenotypes, characterized by delayed hair growth, disorganized hair follicles, dry scaly skin and weight loss. Both mice also show altered epidermis markers differentiation (increased loricrin levels and decreased TGM expression), resulting in immature production of stratum corneum, and accumulation of inflammatory and T cell population. The ADAM17/EGFR axis is therefore involved in skin barrier formation and keratinocyte differentiation (Fig. 7C) [118]. EGFR plays a crucial role in both cancer and immune diseases. EGFR inhibitors (EGFRIs) are used as targeted therapies for cancer, blocking EGFR signaling and reducing tumor growth [119–122]. However, EGFRIs can also induce side effects, including cutaneous complications like rash, papulopustular eruption, xerosis, and pruritus. Interestingly, the onset of skin disorders may be associated with the benefits of treatment and long overall survival in cancer patients [123–126]. The papulopustular eruption is the most frequent EGFRIs reaction which occurs in 79–90% of cases, characterized by erythematous papules and pustules which develop during the early week of treatment [127, 128]. Interestingly, the onset of skin disorders is associated with the benefits of treatment and long overall survival of patients. The study of Joly-Tonetti et al. has reported the different effects of EGFRIs on molecular and functional pathways in keratinocytes, modulating the expression of various markers, including filaggrin, involucrin, desmoglein-1 and Ki67, expressed in the proliferating cells. EGFRIs can increase the expression of involucrin, filaggrin and desmoglein-1, while decreasing cellular proliferation at the basal layers, suggesting an impact on pro-differentiation pathways and skin barrier homeostasis (Fig. 7D) [129]. EGFR also plays a role in wound closure repair and chronic inflammatory disease, such as allergic dermatitis and psoriasis. In these conditions, EGFR positivity correlates with high levels of TGF-α and Granulocyte/macrophage-colony stimulating factor (GM- CSF), involved in the activation of macrophages and immune response [130]. Indeed, high expression of TGF-α leads to EGFR activation and strengthen TNF-α effect, leading to high GM-CSF expression through regulation of activator protein (AP)−1 and NFkB expression. Moreover, TNF-α promotes the phosphorylation of c-Jun involved in the transactivation of AP-1. As a result, in murine models and in human keratinocytes cultures, ablation of EGFR or pharmacological inhibition of ERK affects GM-CSF, ERK 1/2, JNK 1/2 and c-Jun expression and wound repair (Fig. 7E) [131]. In conclusion, EGFR plays a dual role in the regulation of inflammatory process in immune diseases and in the development of cutaneous complications upon EGFRIs chemotherapy.

### Brain development

**Fig. 8:**
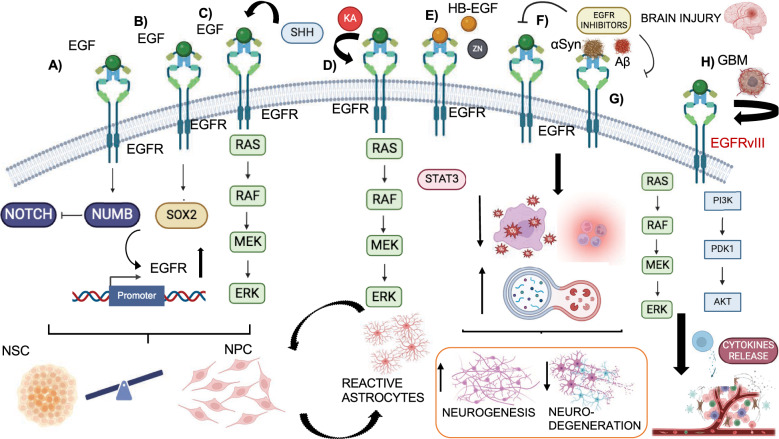
The EGFR role in brain development. This image shows the different roles of EGFR in the brain, including neurogenesis and neurodegenerative diseases and glioblastoma development. **A**–**C** EGFR regulation: **A** The interplay between EGFR and Notch signaling regulates the balance between NSC and NPC. EGFR reprimes Notch through Numb expression, which interacts with E3 ubiquitin ligases degrading Notch and promoting NPC proliferation and expansion. **B** EGFR regulates the expression of SOX2 that, in turn, binds with EGFR promoter leading to EGFR expression and signaling activation. **C** SHH promotes transactivation of EGFR influencing NPC pool proliferation. **D**–**F** EGFR in brain injuries: **D** Kainic Acid (KA) treatment and **E** HB-EGF ligand and zinc induce EGFR expression and MAPK-ERK signaling activation, resulting in the differentiation of NCP into reactive neural stem cells (React-NSCs) that contribute to astrocytes formation, impairing neurogenesis. **F** On the contrary, EGFR inhibition preserves NSC pool, stimulating the neurogenesis process through the migration of neurons towards injured regions and generation of new neurons. **G** EGFR role in neurodegenerative diseases: Pharmacological inhibition of EGFR leads to the decrease of oxidative stress and inflammation and to the increase of the autophagy process involved in the reduction of peptides accumulation. **H** EGFR role in glioblastoma: the inhibition of EGFR affects the downstream signaling pathway activation, decreasing the levels of pro-inflammatory chemokines, such as CCL2 levels, and the infiltration of tumor-associated macrophages involved in the glioblastoma development

Brain development is a dynamic and complex process that begins at the third gestational week and proceed into late adolescence. It involves the proliferation of neural stem cells (NSCs) and neuroblast progenitor cells (NPCs), their differentiation into specific type of neuronal cells and neurons that migrate and occupy the cerebral cortex in the central nervous system (SNC). Here, neurons participate in the branching morphogenesis process, establishing connections (axons and dendrites) and forming an intracellular network for signals transmission [132, 133]. The mature adult brain retains two proliferative niches, derived by the germinal regions of the brain, the subgranular zone (SGZ) and the subventricular zone (SVZ) involved in the neurogenesis and gliogenesis processes that results in new production of neurons and glia, including astrocytes, oligodendrocytes, Schwann cells, and microglia in the adult brain [134]. In these zones Notch and EGFR signaling regulates the balance between NSCs and NPCs, essential to provide and maintain specific neural populations. Studies in mice have demonstrated that EGFR overexpression decreases Notch expression, NSC proliferation and neurospheres number and size, resulting in an expansion of NPC pool; conversely, NICD (Notch1 intracellular domain) overexpression can rescue these effects. EGFR regulates Notch signaling via Numb expression which interacts with E3 ubiquitin ligases to degrading Notch receptor (Fig. 8A) [135]. Thus, the quiescence and inactive mitotic state of SNC is regulated by Notch signaling which induces their self-reviewal and symmetric division of SNCs. EGFR promotes the proliferation and expansion of NPCs, which are the multipotent precursor cells of SNC. A positive loop between the transcription factor, Sox2 and EGFR has been observed in the regulation of the NPC pool. Sox2 binds to the EGFR promoter, activating its signaling pathways, while EGFR activation also promotes Sox2 expression the inhibition of EGFR, PI3K and the Erk1/2 reduces Sox2 expression and NCP colony size (Fig. 8B) [136]. The Sonic hedgehog (SHH) pathway also regulates EGFR mitotic activity on NPCs during late brain development is. SHH inhibition synergistically reduces EGF-responsive stem cell proliferation with EGFR inhibition, while SHH treatment induces EGFR phosphorylation and ERK1/2 activation. SHH inhibition also reduces the levels of GFAP and SOX2, markers of radial glial (RG) cells, suggesting that SHH promotes EGFR signaling in RG target cells (Fig. 8C) [137]. Interestingly, besides RG cells, the expression of EGFR was also found in multiple lineage progenitor cells, including astrocyte progenitor cells (APCs), oligodendrocyte progenitor cells (OPCs), intermediate progenitor cells and immature inhibitory neurons (INs) [138]. EGFR expression occurs during embryogenesis but is reduced in the adult brain, when active central system nervous matures. EGFR is re-expressed in pathological condition such as epileptogenesis and brain injuries. In these cases, EGFR upregulation in quiescent astrocytes leads to reactive astrocyte activation, neurodegeneration, loss of neurons and oligodendrocytes, and demyelination. The study of Pastor-Alonso et al. revealed a high concentration of EGFR in MTLE-HS (mesial temporal lobe epilepsy with hippocampal sclerosis) mice, especially in hippocampal NCP cells, compared to control mice, after Kainic Acid (KA) injection which is a neuroexcitatory amino acid agonist functioning by activation of glutamate receptor. EGFR regulates, through activation of phospho-STAT3 and phospho-ERK1/2, the differentiation of NCPs into reactive neural stem cells (React-NSCs), which contribute to astrocytes formation and impair neurogenesis (Fig. 8D). HB-EGF ligand and zinc treatment activate EGFR, promoting NPC proliferation and React-NSCs induction (Fig. 8E). Blocking EGFR pathway represents a potential molecular target to preserve NSC pools [139]. Pharmacological inhibition of EGFR has shown promise in brain injuries repair, stimulating the migration of neurons towards damaged regions. EGFR overexpression, induced by its ligand TGF-α, expressed in microglia cells in response to brain injury, promotes neuroblast proliferation in the SVZ, as evidenced by increase of Ki67. Inhibition of TGF-α release or of EGFR expression can stimulate progenitor SVZ migration into the damaged zone and new neurons generation (Fig. 8F) [140]. Different inhibitors of the EGFR downstream protein cascade, such as PI3K/AKT, MEK/ERK and mTOR, have NPC properties. Neurosphere cultures of NSPCs have shown that, while all three pathways contributes to NPC proliferation, the inhibition of MEK/ERK increases the expression of the astrocyte marker GFAP and of the neuronal marker bIII-tubulin, influencing NPC differentiation and inhibition of mTOR impacts NPC survival [141]. Neurodegenerative disorders are characterized by formation of accumulated aggregates of misfolded or mutated proteins/peptides, such as amyloid-beta (Aβ) peptides in Alzheimer’s disease (AD), α-synuclein (αSyn) in Parkinson’s disease (PD), and mutated huntingtin (mHtt) in Huntington’s disease (HD); aggregate formation is associated with cognitive and physical deficit of dementia [142]. Single nucleotide polymorphisms (SNPs) of EGFR are associated with increased risk of AD and PD [143, 144]. EGFR could be activated by pathogenic molecules and proteins involved in these diseases, contributing to their progression. Concerning PD, in vitro experiments showed that EGFR downregulation reduces αSyn levels and that αSyn treatment induces EGFR expression. In vivo experiments using a mice model of αSyn propagation, the inhibition of EGFR via AZD3759 reduces phosphorylation of EGFR and cellular uptake of αSyn, ameliorating the pathology. Interestingly, AZD3759 increases the levels of the autophagy markers LC3-II/LC3-I and decreases p62 expression, suggesting a neuroprotective role of autophagy, which is also demonstrated in AD [145, 146]. In AD, an altered cleavage of Amyloid precursor protein (APP), a type 1 integral membrane glycoprotein expressed in the brain, manipulated by Presenilins (PS)/γ-secretase leads to formation of insoluble amyloid-β (Aβ40–42) that deposit as pathological plaques in brain [147]. In APP/PS1 double transgenic mice, characterized by presence of aggregates Aβ peptides, an accumulation of activated EGFR in hippocampal tissues compared to control mice has been observed, associated to memory loss which is rescued upon EGFR inhibitor treatment for long time. The mechanism is due to Aβ peptides production that act as ligands of EGFR, leading to its activation and neurodegeneration progression [148, 149]. Finally, a recent study by Mansour et al. reported the effect of dual inhibition of EGFR and HER2 with lapatinib ditosylate (LAP) in the treatment of AD. As results, mice with cognitive decline showed a decrease of oxidative stress, neuroinflammation, reactive astrocytes and increase of autophagy associated to HER2 inhibition, leading to reduction of Aβ peptides accumulation [150]. Moreover, LAP increases the expression of the PI3K/Akt/GSK-3β pathway, counteracting neuronal apoptosis and axon degradation due to oxidative stress-EGFR induction (Fig. 8G) [151]. EGFR is also involved in glioblastoma (GBM) development [152]. Targeting EGFR and its downstream pathways, including c-MET and mTOR, has shown potential in improving survival in GBM patients. The EGFRvIII mutation, commonly expressed in GBM patients, is a truncated form of receptor that induces constitutive activation of EGFR [153]. In U87-H GBM cells, EGFRvIII expression correlates with activation of PI3K and c-MET receptor pathways. The inhibition of c-MET overcomes the EGFRvIII-mediated resistance to chemotherapeutics, modulating apoptotic pathway including Bcl-XL and caspase 3 [154]. Moreover, the expression of EGFR and EGFRvIII in GBM leads to an increase of macrophages recruitment through KRAS activation, which upregulates the levels of chemokine CCL2, contributing to immunosuppression [155]. The combination of erlotinib and MLN0128, an mTOR inhibitor, affects p-EGFR, p-AKT, p-ERK, and p-RAS40 levels and as well as CCL2 levels and other pro-inflammatory chemokines, decreasing the infiltration of tumor-associated macrophages and prolonging the survival of GBM mice (Fig. 8H) [156].Poor survival is linked to high expression of PDL-1 and a low CD8+ T cell infiltration. EGFR-ERK signaling stabilizes PDL-1 expression, suggesting that the modulation of the tumor microenvironment (TME) could represents an alternative strategy to impairs GBM progression [157, 158]. Altogether, these data highlight that EGFR inhibition, through monoclonal antibodies or TKI combined with other chemotherapeutics could be a potential and prominent therapeutic strategy in the treatment of neurodegeneration and neuronal pathological diseases, including GBM progression.

### T cell regulation

**Fig. 9:**
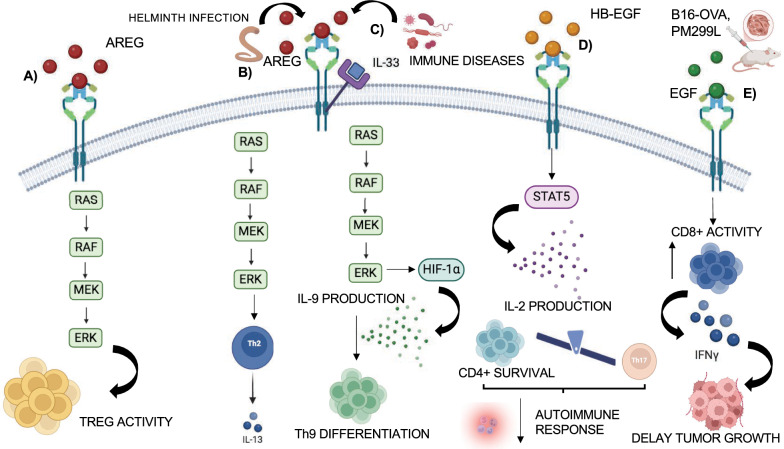
The EGFR role in T cell regulation. **A** EGFR role in Tregs activity: AREG-EGFR binding leads to activation of MAPK signaling that enhances the suppressive activity of Treg cells. **B**–**D** EGFR role in T helper cells activity: **B** Following gastrointestinal helminth infection, activated EGFR in CD4+ helper T forms a complex with IL-33R, leading to IL-13 secretion and Th2 expression. **C** A similar pathway promotes the differentiation of Th9 cells. The binding of AREG-EGFR, boosted by IL-33 exposure, promotes the activation of EGFR signaling pathway, including the activation of HIF1α that binds IL9 promoter, resulting in IL-9 production and enhancement of Th9 cell differentiation. **D** The binding of HB-EGF to EGFR causes STAT5 expression, that leads to IL-2 production involved in the survival of CD4+ cells, at the expense of Th17 cells differentiation. **E** Engineered CD8 + lymphocytes expressed EGFR in mice bearing B16-OVA (melanoma murine cell lines) and PM299L (hepatocarcinoma cell lines) tumors. Upon EGF ligands, CD8+ T cells respond better to EGFR signaling activation, producing more IFN-γ and leading to a delay in the tumor growth

The immune system represents the main defense of the human body against pathogens and harmful cells, and consists of the innate and the adaptive systems. The adaptive immune system involves B cells and T cells. B cells secrete antibodies which bind to specific antigen, blocking the binding of pathogens to host cells, and present the antigen to T cells. T cells, through their T cell receptor (TCR), recognize the antigen complex with a host molecule called the major histocompatibility complex (MHC) and initiate a cascade of downstream signaling, including secretion of chemokines and cytokines to attract and induce activation of more naïve T cells, leading to apoptosis of infected cells [159]. T cells are divided into and CD8+ cytotoxic T cells that kill directly infected cells, CD4+ helper T cells that send signals to other immune cells to fight the invaders and T regulatory cells (Tregs) that play an immunosuppressive role, preventing T cells from attacking body self-antigens, avoiding autoimmune disease. The migration of Tregs to tissues and inflamed site is crucial for appropriate immunosuppressive activity [160]. EGFR is a factor that controls Treg activity under inflammatory conditions. EGFR is also expressed in hematopoietic cells, including macrophages, mast cells, plasma cells, monocytes and lymphocytes, suggesting a regulatory role of EGFR in the immune response. EGFR is expressed in Tregs upon activation, and its action is mediated by the AREG ligand. AREG-deficient mice exhibit impaired Treg function upon infection or bone marrow (BM) transplantation. AREG prolongs EGFR signaling (MAPK kinase activation), enhancing Treg suppressive activity (Fig. 9A). The interaction between mast cells and Tregs, with mast cells as sources of AREG, enhances Treg function. Moreover, given the EGFR contribution to the immunosuppressive environment, clinical EGFR inhibitor could improve chemo-therapeutics treatments of tumors, reducing Treg activity and improving the anti-tumor immune response [161].

AREG/EGFR signaling is also activated in Th helper cells. After gastrointestinal helminth infection, activated CD4+ helper T cells in mice show increased EGFR expression, induced by cytokine-dependent signaling. EGFR deficiency in T cells increases susceptibility to helminth infection. Activated EGFR forms a complex with T1/ST2 (IL-33R) that induces IL-13 secretion by activated Th2 cells, following exposure to IL-33 and activation of MAPK signaling (Fig. 9B) [162]. A similar pathway involving AREG-EGFR and IL-33 exposure is important for the differentiation of Th9 cells, which produce IL-9 and have anti-tumor and anti-inflammatory properties. TGF-β1 and IL-4 drive the differentiation of naïve CD4+ T cells into Th9 cells. AREG and EGFR expression in Th9 cells, along IL-33 exposure, promote EGFR signaling and IL-9 production, driven by HIF1α that binds IL9 promoter, resulting in enhancement of Th9 cell differentiation (Fig. 9C) [163].

HB-EGF expression is also important for the CD4+ helper T cells activation, and prevents their differentiation in Th17 cells, which are involved in the autoimmune response. HB-EGF-deficient CD4 T cells show increased Th17 cells, leading to inflammatory and wasting diseases, which are likely due to expression of TGFβ at the expense of HB-EGF/EGFR/STAT5 signaling and IL-2 production, essential to promote survival of CD4+ cells and avoid Th17 differentiation [164]. Thus, EGFR plays a critical role in maintaining CD4+ cell homeostasis, promoting T cell proliferation, activation and cytokines production. Pharmacological inhibition of EGFR impairs the activity of CD4+ cells (Fig. 9D). For this reason, atherosclerosis, a chronic inflammatory disease due to autoimmune response in which CD4+ T lymphocytes act as pathogen cells, can be treated with EGFR inhibitors**.** HB-EGF has been found in human atherosclerotic plaques: EGFR inhibition leads to a reduction of T cell migration and cytokines expression, limiting atherosclerosis development [165].

Lozano et al. modified CD8+ cytotoxic T cells with a retrovirus expressing EGFR and GFP, observing different effects in *vitro* and in *vivo.* EGFR-Engineered CD8+ lymphocytes showed enhanced proliferation and IFN-γ and TNF-α production, compared to control cells. Transfer of EGFR-Engineered CD8+ lymphocytes in mice bearing B16-OVA (melanoma murine cell lines) and PM299L (hepatocarcinoma cell lines) tumors, led to increased IFN-γ production and delayed tumor growth, respectively, suggesting their potential anti-tumor activity (Fig. 9E) [166].

## EGFR role in organ-specific branching morphogenesis

Branching morphogenesis is a dynamic and evolutionary process that occurs during embryogenesis and creates ramified and arborized structures in different organs, such as the pancreas, kidney, mammary gland, and lung. Branching morphogenesis is a process that generates a bi- or trifurcation of multi-layered epithelial tips into the surrounding mesenchyme, spatially and temporally coordinated by the presence of several signaling molecules and growth factors that establish epithelial-mesenchymal tissue interactions. Each organ adopts different branching strategies that lead to its final structure and architecture [167, 168]. In this Section, we will describe role of EGFR in the development of different organs, characterized by branching morphogenesis process.

### Pancreas development

In the pancreas, branching morphogenesis leads to the formation of two main compartments: the exocrine pancreas, which produces digestive enzymes, and the endocrine pancreas, which contains clusters of cells called islets of Langerhans, which are characterized by four endocrine cell types, such as insulin-producing β-cells, glucagon-producing α-cells, somatostatin-producing δ-cells, and pancreatic polypeptide–producing PP-cells. The organogenesis and development of the pancreas are controlled by mesenchymal-epithelial interaction, secretion of several growth factors, and expression of transcriptional factors [169–171].

EGFR plays a critical role in both fetal and postnatal pancreas development, particularly in the coordination of β-cell proliferation and differentiation [172–174].

**Fig. 10:**
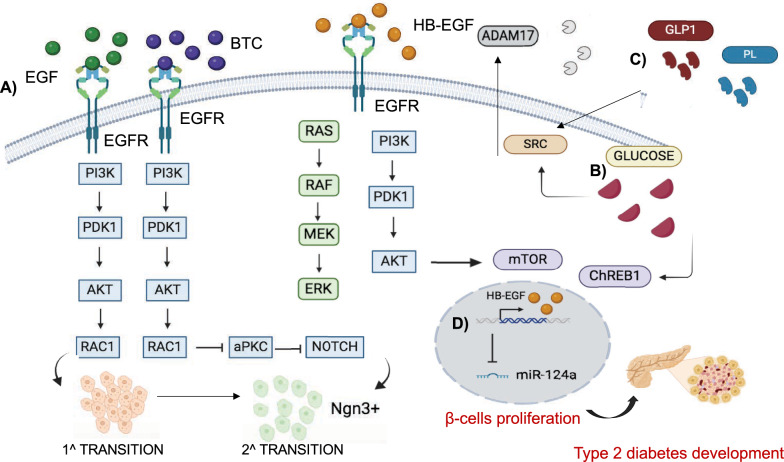
The EGFR roles in fetal and postnatal pancreas development. **A** β-cell commitment and differentiation: Dynamic cell polarity changes are regulated by EGFR, which drives β-cell commitment. It depends on the different EGFR ligands. EGF binds EGFR and activates PI3K and RAC1 proteins, which reduce apical polarity induction in the early pancreatic progenitor epithelium, leading to endocrine commitment towards a β-cell fate (first transition). Upon BTC-EGFR binding, RAC1 inhibits αPKC, which leads to downregulation of Notch signaling and activation of Ngn3, involved in β-cell differentiation (second transition). **B** β-cell proliferation: Excess of nutrients as glucose induces ChREBP expression, that promotes HB-EGF transcription and SRC kinase. SRC kinase triggers EGFR transactivation via ADAM metalloproteases. As a result, HB-EGF binds EGFR and activates its signalling cascades, including MAPK, PI3K/AKT and mTOR, leading to increase of cellular transcription and β-cell proliferation. These events are associated to diabetes development. **C** EGFR transactivation via hormones such as GLP1 and Placental lactogen (PL). **D** miR-124a acts as negative regulator of β-cells proliferation and insulin resistance. EGFR signalling activates, through ERK and AKT downstream signalling pathways, the transcription factor ETS2, which represses miR-124a, leading to expression of genes involved in insulin secretion and control β-cells development

During fetal development, EGFR signalling influences the commitment and differentiation of pancreatic progenitors towards β-cell fate. In 3D fetal cell cultures, EGF treatment induces cellular proliferation at the expense of pancreatic differentiation. On the contrary, the absence of EGF notably reduces cellular expansion, increasing the expression of mature acinar and endocrine cell markers [175]. In organogenesis, cells undergo a morphogenetic process through changes in their architecture or polarity to define their specific behaviour. The epithelial cells maintain their apical-basal polarity to play different functions, including creating a barrier against pathogens and large molecules, promoting exocytosis process of several cargos, and organization of complex structures such as tubes or their domains [176]. The cell polarity is controlled by apical, lateral, and basolateral polarity factors and focal adhesion interaction with the ECM. The alteration of these processes leads to dynamic polarity changes based on the epithelial-mesenchymal transition and differentiation processes [177]. In this regard, the apical polarity of pancreatic cells is regulated by EGFR, which participates in β-cell commitment. This process includes two transitions where EGFR regulates different signalling pathways, which showed a specific outcome depending on the EGFR ligands. In the first transition, the ligand EGF binds EGFR and activates PI3K and RAC1 proteins, which reduce apical polarity induction in the early pancreatic progenitor epithelium, leading to endocrine commitment towards a β-cell fate. In the second transition, the ligand BTC binds EGFR more efficiently than EGF, inhibiting αPKC at the apical domain upon PI3K and RAC1 activation. αPKC is the main effector of apical polarity downregulated by RAC1 protein. The decrease of αPKC leads to downregulation of Notch signalling [178], activating Ngn3 involved in the β-cell differentiation (Fig. 10A) [179].

The role of EGFR has also been investigated in adult β-cell proliferation, using two different mouse models, generated by surgical removal of part of the pancreas (PPx) and excess of nutrients, respectively. Both processes induce a local inflammation and EGFR upregulation that stimulates β-cell proliferation through different mechanisms. On the one hand, EGFR increases the expression of cyclin D1 and downregulates p27 gene, involved in the cell cycle, to promote β-cell proliferation [180]. On the other hand, EGFR regulates β-cell proliferation via EGFR–mTOR signalling in the presence of HB-EGF ligand. An excess of nutrients, such as in glucose treatment, regulates in two ways the upregulation of HB-EGF: by action of ChREBP, which promotes HB-EGF transcription; and by induction of SRC kinase that triggers EGFR transactivation via ADAM metalloproteases. As a result, HB-EGF triggers downstream EGFR signaling cascades, including MAPK, PI3K/AKT, and mTOR pathways that are involved in β-cell proliferation and mass increase (Fig. 10B) [181]. Of note, the excess of nutrient leads to type 2 diabetes development characterized by insulin resistance and a first compensatory response of β-cell, which results in an increase of its proliferation and insulin secretion, which later drives β-cell exhaustion [182]. Therefore, increasing β-cell proliferation is a therapeutic strategy for diabetes treatment, and the upregulation of EGFR could be crucial for the improving β-cell function [183, 184]. A possible pharmacological approach via EGFR regulation could be based on Exendin-4. Exendin-4 is an analogue of the hormone GLP-1 that binds the GLP-1 receptor, a member of the G protein-coupled receptor (GPCR) superfamily. GLP-1 regulates β-cell proliferation, insulin secretion and synthesis through EGFR transactivation, mediated by SRC kinase and metalloprotease expression (as previously described), resulting in the PI3K-AKT and PCK protein activation. Upon EGFR deletion, the effect of Exendin-4 on β-cell and islet size, β-cell proliferation, and insulin content is decreased, causing development of glucose intolerance and supporting the cross-talk between Exendin-4/GLP-1 and EGFR [185]. Besides GLP1, the hormone Placental lactogen (PL) is also involved in the transactivation of EGFR and consequent β-cell proliferation during pregnancy. Upon binding to its receptor PRL-R, PL induces EGFR transactivation that leads to upregulation of downstream ERK, AKT and mTOR pathways. Altogether, they stimulate surviving gene expression that is required for beta-cell expansion, favouring an increase of islet number during pregnancy (Fig. 10C) [186]). Contrary to the EGFR effect, miR-124a—overexpressed in the islet of diabetic patients—determines a dysfunction of β-cell, targeting the expression of the glucose-sensing regulator forkhead box a2 (FOXA2), NEUROD1, and PDX genes, related to insulin biosynthesis and secretion. Consequently, the negative regulation of miR-124a could be another potential strategy to overcome insulin resistance. EGFR is a negative regulator of miR-124a: EGFR signalling activates, through ERK and AKT downstream signalling pathways, the transcription factor ETS2, which represses miR-124a, leading to the expression of genes involved in insulin secretion and control β-cells development (Fig. 10D) [187].

In summary, EGFR plays a dual role in pancreas development, regulating fetal β-cell differentiation and adult β-cell proliferation. In the first case, EGFR regulates the proliferation and differentiation of endocrine progenitors, determining lineage differentiation via cell polarity changes. In the other case, EGFR promotes the upregulation of β-cell proliferation as a response after inflammation due to injuries, including surgical pancreas remotion or high-fat diet that promotes insulin resistance, suggesting its function as a therapeutic diabetes target.

### Kidney development

**Fig. 11:**
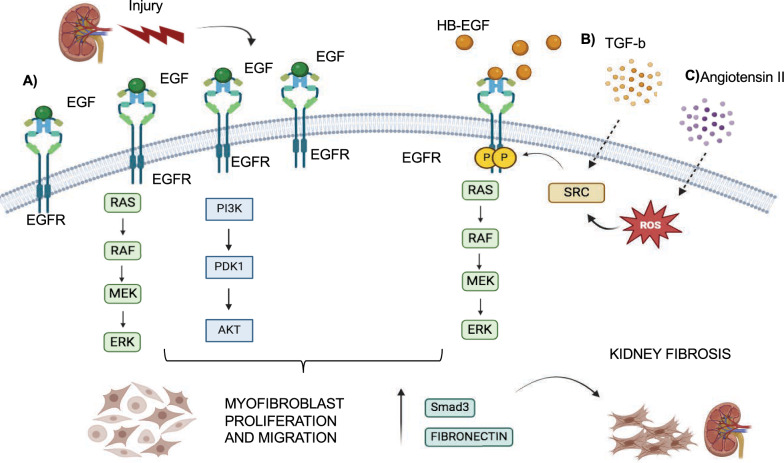
The EGFR roles in kidney fibrosis. **A** Kidney injury increases EGFR levels in the epithelial renal cells. EGFR signalling activation induces cellular proliferation and migration of myofibroblasts toward the sites of the injury. **B**, **C** EGFR transactivation can be caused by different proteins such as TGF‐β and Angiotensin II. TGF‐β acts through the activation of SRC kinase that phosphorylates EGFR, leading to persistent activation of EGFR; Angiotensin II increases ROS production that mediates SRC kinase activation. EGFR transactivation promotes Smad3 and fibronectin upregulation. This EGFR downstream signalling cascade favours the development of fibrosis disease

The urinary system, including kidneys, ureters, bladder, and urethra, plays an essential function of the organism, i.e., filtering, storing and removing liquid waste from the body. Kidneys represent the principal unit of the urinary system. They regulate the balance between water and minerals (including electrolytes) in the body, filtrate waste products from blood, secrete hormones and control blood pressure. Kidney development occurs via mesenchymal-epithelial interactions regulated by different growth factors and signalling pathways that are involved in the branching morphogenesis process [188]. Among these factors, EGFR ligands participate in stimulating tubulogenesis and branching morphogenesis in vitro, suggesting a role of EGFR in kidneys development [189, 190]. EGFR is widely expressed in the mammalian kidney cells such as podocytes, endothelial cells and mesangial cells, as well as medullary interstitial cells and in the glomerulus and in multiple tubule segments, including proximal and cortical tubule, loop of Henle, medullary and distal collecting duct [191]. Under physiological conditions, the most important function of EGFR in kidney is the regulation of renal electrolyte homeostasis and store-operated Ca^2+^ channel [192]. Altered expression of EGFR has been observed in interstitial fibrosis, which represents one of the most common features of chronic kidney disease (CKD). After injury, EGFR levels increase in the epithelial renal cells, interstitial cells and myofibroblast cells. Here, the function of EGFR is inducing the proliferation and migration of myofibroblasts toward the sites of the injury and regulating the expression of genes associated to myofibroblast differentiation and fibrogenesis. Accordingly, the depletion of EGFR in the pericytes correlates with a decrease of proliferation and migration of myofibroblasts and consequent decrease of kidney fibrosis (Fig. 11A). Although the canonical pathway of EGFR activation is the most frequent way through which the receptor plays its function, EGFR also interacts with other factors that induce its transactivation and activate different signalling pathways. Among these factors, the cytokine Transforming growth factor‐beta (TGF‐β) can induce the activation of its downstream Smad signalling pathways by EGFR transactivation [193]. Of note, TGF‐β regulates the secretion of matrix proteins such as collagen and fibronectin by mesangial cells (MCs), which are the main producers of glomerular ECM. The correlation between EGFR and TGF‐β observed in kidney fibrosis suggests a cross-talk between them. Simultaneously to canonical ligand HB-EGF-EGFR activation that induces short signalling, TGF‐β mediates EGFR transactivation, which is associated to prolonged signals, through the activation of SRC kinase. SRC kinase phosphorylates EGFR at different sites, compared to canonical autophosphorylation, leading to persistent activation of EGFR that promotes phosphorylation of Smad3 and fibronectin upregulation. This EGFR downstream signalling cascade favours the development of fibrosis disease (Fig. 11B) [194]. However, another type of cross-talk between EGFR and TGF‐β has been observed, mediated by Angiotensin II, a proinflammatory cytokine. Angiotensin induces EGFR transactivation, which leads to the increase of ROS production that mediates SRC kinase activation. The latter promotes the persistent activation of EGFR, TGF‐β and Smad activation, causing fibrosis development [195]. Angiotensin II-dependent transactivation does not occur when the EGFR ligand HB-EGF is absent (Fig. 11C). Under this condition, reduced inflammation and renal injury can be observed, suggesting that HB-EGF is crucial to mediate Angiotensin II-EGFR binding [196]. All these mechanisms evidence the involvement of EGFR, via canonical or non-canonical activation, in the kidney fibrosis development, through the expression of ECM proteins by TGF‐β, suggesting that targeting EGFR might be a potential strategy to inhibit TGF-β in kidney fibrosis. Accordingly, the dual treatment Losartan + Erlotinib strongly attenuates fibrogenesis, preventing the interstitial expansion and the expression of genes involved in the extracellular matrix production. Losartan is an Angiotensin II receptor blocker that impedes the binding of Angiotensin to receptor AT1, while Erlotinib is an EGFR-specific tyrosine kinase inhibitor (TKI). On the basis of their function, the dual treatment leads to a decrease of EGFR phosphorylation and of EGFR and TGF-β expression, correlated to the reduction of fibrosis alteration [197, 198] and may represent a possible new target therapeutic strategy against kidney fibrosis. As previously reported, ROS production is a consistent step during EGFR transactivation that leads to the prolonged EGFR signals and activation of TGF-β pathways, causing kidney fibrosis. At the same time, a relation between EGFR and ROS levels has been observed in the progression of diabetic nephropathy, a kidney disease due to abnormality and microvascular complications in diabetic patients that leads to the dysregulation of many homeostatic signalling pathways. Interestingly, this disease shows a different mechanism through which EGFR can lead to endoplasmic reticulum (ER) stress. The accumulation of misfolded proteins in the ER is defined ER stress. It increases ROS production and activates an adaptive response, the unfolded protein response (UPR). The function of UPR is to restore ER homeostasis, increasing the folding efficiency by upregulating molecular chaperones and reducing the translation of most proteins to prevent ER workload and further unfolded proteins. Under prolonged ER stress, UPR can lead to cell death [199]. The increase in ER stress, ROS level and EGFR protein observed in the diabetic nephropathy has suggested a possible link between these signalling pathways. As a result, besides TGF-β, the pharmacological inhibition of EGFR also reduces AKT activation, ROS production and UPR gene expression, decreasing kidney fibrosis [200]. EGFR may control ER stress signalling mediating AKT activation, which is associated to the regulation of genes related to ER stress [201].

### Mammary gland development

**Fig. 12:**
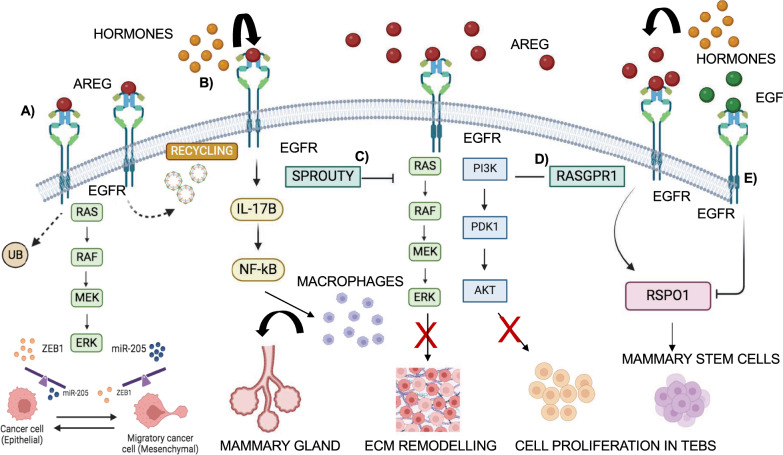
The EGFR roles in mammary gland development. **A** EGFR role in the switch between the EMT and MET: the balance between EMT and MET represents one of the important processes for mammary gland development, regulated by EGFR signalling. EGF binds EGFR, and promotes p-ERK activation, which increases the expression of mesenchymal marker ZEB1 and decreases the expression of the epithelial marker miR-205, favouring mesenchymal phenotypic changes. Upon activation, EGFR undergoes ubiquitination and degradation, reducing EGFR expression on plasma membrane. On the contrary AREG, binds EGFR and induces a weak activation of p-ERK and increases the expression of miR-205 at the expense of ZEB1, reverting the cells characteristic from mesenchymal to epithelial phenotype. AREG-EGFR binding leads to EGFR recycling back to the plasma membrane and, consequently, prolonged signal activation. **B** Hormones levels and EGFR activation: hormones stimulation increases the expression of AREG, which binds EGFR. This leads to IL-17B upregulation, which induces NF-κB dependent cytokine expression, responsible for macrophages infiltration in the mammary gland duct. **C–E** EGFR regulation: **C** Sprouty and **D** Rasgrp1 are negative regulators of EGFR. The former inhibits EGFR-MEK-ERK signalling, impairing ECM remodelling, collagen deposition and cellular migration; the latter inhibits EGFR-AKT axis regulation, impairing mTOR signalling and TEBs cellular proliferation during late stage of puberty. **E** Upon hormones stimulation, AREG positively regulates R-spondin expression, while EGF expression negatively regulates Wnt and R-spondin, that are involved in mammary stem cell (MaSC) self-renewal

The mammary gland is a crucial organ for milk production in female mammals. Its development occurs in distinct stages, with branching morphogenesis during puberty being a critical step. During puberty, terminal end buds (TEBs) at the leading edge of duct, progress into the mesenchymal mammary fat pad where they undergo an elongation process. Then, by bifurcation, TEBs give rise to new ducts forming a specific ductal system, which is important for later mammary development and function. Then, in the adult gland, during pregnancy and in response to lactogenic hormones, epithelial cells proliferate and form alveolar structures that are crucial in the production of milk, which depends on the ECM cues. The ECM, containing metalloproteinases and their inhibitors, receptors and integrins, separates the epithelium compartment from stromal cells, supports the mammary gland structure, helps milk production and drives the ducts orientation [202]. Cellular proliferation and ECM remodelling are the most important events occurring in the mammary gland branching morphogenesis. These processes are governed by changes in the epithelium and in growth factors and hormones expression and epithelium-stroma interactions [203]. During postnatal development, a key interaction occurs between estrogen production, AREG expression in the mammary epithelium and EGFR activation in stromal cells where it is mostly expressed. AREG, a ligand of EGFR, is downregulated during and after pregnancy, while it is upregulated during puberty, when cellular proliferation and duct elongation occur in order to promote duct morphogenesis. The first evidence that AREG deletion in mice affects mammary gland morphogenesis [204] has suggested a link between estrogen production and mammary gland development mediated by activation of the AREG-EGFR axis. The upregulation of AREG upon estrogen production, due to increased ADAM17 expression, leads to its EGFR binding only in stromal cells, where EGFR is expressed [94–96]. Such ligand binding induces the expression of matrix metalloproteinases such as MMP2, MMP3 and MMP14, involved in the ECM remodelling necessary for functional differentiation of the mammary gland [205]. Contrary to AREG expression, the other ligands, such as EGF and TGF-α, show a constant level during mammary development. The binding of different ligands to the EGFR receptor leads to the activation of specific downstream pathways and opposed EGFR fates (degradation or recycling), which causes different effects on the epithelial mammary cells, such as proliferation, differentiation, epithelial-mesenchymal transition (EMT), and, reversely, mesenchymal-epithelial transition (MET). The balance between these processes is crucial for mammary gland development. An uncontrolled EMT process plays a key role in cancer formation. Thus, the switch between the EMT and MET process must be highly controlled. The regulation of EMT and MET depends on the ligand affinity and its ability to determine EGFR fate and downstream signalling pathways. EGF binds with high affinity EGFR, promotes p-ERK activation which increases the expression of mesenchymal marker ZEB1 and decreases that of the epithelial marker miR-205, favouring mesenchymal phenotypic changes. On the contrary, AREG-EGFR binding induces a weak activation of p-ERK and increases the expression of miR-205 at the expense of ZEB1, reverting the cell phenotype from mesenchymal to epithelial (Fig. 12A) [206]. Furthermore, proliferation and differentiation are the other two important processes for mammary gland development regulated by EGFR ligands [207]. As for EMT processes, the EGFR fate and the activation of downstream pathways depend on the affinity of ligands which direct the specific cellular differentiation process. Low-affinity AREG binding promotes luminal lineage, while high-affinity TGF (as EGF) leads to myoepithelial differentiation, due to sustained activation of the EGFR-MEK-ERK pathway [208]. Hormones (estrogen and progesterone) stimulation, besides regulating EGF expression, lead to the increase of macrophages and eosinophils in the stroma, playing an essential role in ductal morphogenesis [209, 210]. The recruitment of this cellular population into the TEBs is mediated by AREG-EGFR signalling pathways. It regulates the expression of chemokines and cytokines as IL-17B, which induces NF-κB dependent cytokine expression, responsible for the macrophage infiltration in the mammary gland duct (Fig. 12B) [211]. Taken together, these data highlight the importance of the EGFR signalling pathways in stromal cells to mediate signals in epithelium compartment in order to influence the postnatal development of the mammary ductal tree. Since EGFR regulates several pathways such as proliferation, differentiation, migration and invasion, its negative regulation could be important to control tumorigenesis process. If, on the one hand, hormones and different ligands are inducers of EGFR, on the other hand, members of the Sprouty family (Spry), expressed in the stromal cells and encoding for intracellular RTK signalling inhibitors, are negative regulators of EGFR signalling in the mammary gland stroma. The loss of Spry1 leads to upregulation of the EGFR–MEK–ERK signalling and the increase of different phenotypes, including branching morphogenesis, epithelial cyst formation, collective migration in in vitro experiments, expression of ECM-remodelling enzymes and collagen deposition and as well as epithelial cellular invasion (Fig. 12C). These phenotypes can be explained by (1) the expression of EGFR in stromal cells where, as described above, it regulates the expression of ECM-remodelling enzymes and (2) its effect on epithelial cells, by activation of paracrine signals, including expression of the FGF ligand that, binding FGF receptors in epithelial cells, promotes cellular migration and invasion [212]. At the same time, since EGFR activates other downstream proteins, as PI3K-AKT, involved in the regulation of cellular proliferation, the EGFR regulation can be modulated by different factors that play a role in specific EGFR-dependent pathways. Among these genes, Rasgrp1 acts in the EGFR-AKT axis regulation, and its deletion in mammary epithelial cells increases the expression of AKT proteins and mTOR signalling, which are involved in protein synthesis, cell proliferation, cell metabolism and survival. Consequently, an increase of TEBs cellular proliferation occurs, which persists in the late stage of puberty, causing a reduced lengthening of the ductal tree (Fig. 12D). At the same time, EGF expression negatively regulates Wnt and R-spondin (agonists of the Wnt/β-catenin signalling pathway) signals that are involved in mammary stem cell (MaSC) self-renewal. On the contrary, AREG expression under hormone conditions (as reported above) positively regulates R-spondin expression (Fig. 12E) [213]. EGFR signalling plays a multifaceted role in mammary gland development, influencing branching morphogenesis, cell differentiation, and ductal morphogenesis. The interplay between hormones, EGFR ligands, and negative regulators ensures a carefully controlled process. Dysregulation of this system can contribute to tumorigenesis. Understanding these complex interactions is vital for future research into mammary gland health and disease.

### Lung development

Lungs are the essential organs of the respiratory system, located in the thoracic cavity and protected by two inner and outer thin tissue layers, the visceral and parietal pleura, respectively. Structurally, lungs are derived from the endoderm gut, which promotes the formation of different tissues along the anterior–posterior axis, via expression of signaling molecules [214].

Lung development occurs through two processes, i.e., branching morphogenesis and alveolarization. The former consists of dividing the trachea and bronchi into secondary and tertiary bronchial branches that ramify into small bronchioles, terminal bronchioles, and respiratory bronchioles. Many factors are involved in the regulation of lung branching morphogenesis, which, through the balance between epithelial proliferation and cell differentiation, leads to the formation, elongation and final arrest of lung bud with consequent tip bifurcation [168]. The activation and inhibition of these factors, as well as their localization in the mesenchyma or in the airway epithelium, is crucial for lung development: mesenchymal growth factors influence the epithelial cellular proliferation to the edge of branch tips. Following branching morphogenesis, alveolarization occurs during the last step of human and mouse lung development after birth. It includes a septation process of the terminal sacs in mature alveoli, which represent the principal surface for gas exchange, surrounded by the capillary network [215, 216]. Alveoli are covered by type 1 (AT1) and type 2 (AT2) epithelial cells, called pneumocytes, that derive from distal progenitors. AT1 cells represent 95% of the peripheral lung surface and are essential for gas exchange; AT2 cells are the precursors of AT1 cells and secrete components of surfactant proteins and regulate the regeneration and repair of lung tissue [217, 218]. The other epithelial cells that populate the epithelium derive from proximal progenitors and include neuroendocrine cells, globet cells, ciliated cells, basal cells and Club cells [219]. These epithelial cells display a physical and functional protective barrier against external pathogens, activating a mechanism of host defense based on the recruitment of mediators of leukocytes [220]. The epithelial cells communicate with vascular endothelial cells and different lineages of mesenchyme cells, such as myofibroblasts (SCMFs), matrix fibroblasts, lipofibroblasts, and mesenchymal alveolar niche cells (also known as MANCs) constituting a regulatory network of signaling pathways that drive the development of alveoli [221]. Several studies have characterized the role of mesenchymal cells in the regulation of epithelial cell proliferation [222] and alveolar regeneration after injury [223], through the interaction with molecular signaling of branching morphogenesis, as the FGF, SHH, BMP and WNT pathways.

EGFR is highly expressed in AT2 cells and represents one of the most crucial players that drive branching morphogenesis. As previously reported, the deletion of EGFR in the embryo and newborn pups affects branching morphogenesis and alveolarization processes, leading to the reduction of bronchi branches formation and sparser bronchial trees surrounded by abundant mesenchyme and irregular, hemorrhagic, collapsed or thick-walled alveoli [224]. The impairment of the surfactant proteins expression supports the defects in the alveolarization process. Among the EGFR ligands, EGF plays an important function in lung branching morphogenesis in cultured normal lungs; however, its exogenous expression in knock-out EGFR mice does not rescue the distressed phenotypes, suggesting that the EGF-EGFR axis is crucial for both branching morphogenesis and alveolarization [224, 225]. The dynamic mechanisms through which EGFR plays its role in lung development are not clear yet. However, mathematical simulations that calculate spatiotemporal interactions of morphogens have recently confirmed the crosstalk between EGFR and macropinocytosis in regulating cellular migration during branching morphogenesis. The macropinocytosis is a clathrin-independent endocytosis that occurs through the formation of irregular vesicles, i.e., macropinosomes, containing extracellular fluid, membrane and solutes. This process is important for antigen presentation, nutrient uptake, recycling of plasma proteins, cell signaling and migration. As a result, macropynocytosis has a significant impact on the physiology of cells and also on tumor growth and cancer cells survival, supplying them with amino acids and nutrients [226–229]. EGFR represents one of the RTK receptors that stimulate macropinocytosis through the PI3K–AKT–RAS signaling pathways, leading to actin cytoskeletal organization and polymerization involved in the macropinosomes formation [230]. In light of these data, since EGF is highly expressed at the tip of branches as well as in the macropinocytosis induction, the driving force of the branching formation is represented by nutrients uptake due to macropinocytosis stimulation upon EGFR activation [231].

**Fig. 13:**
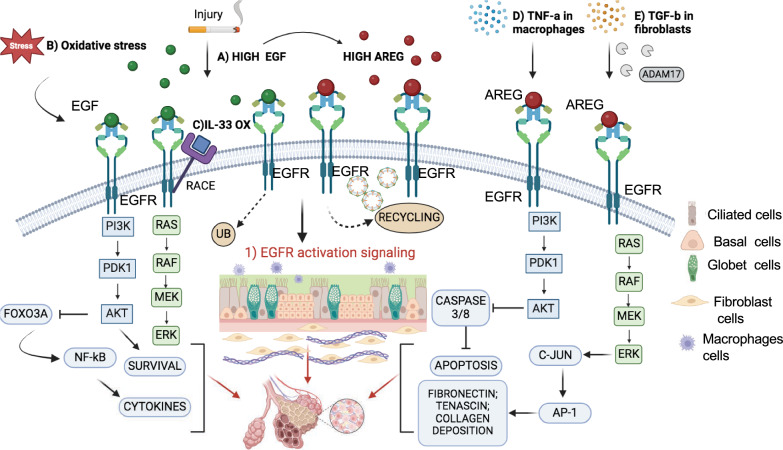
The EGFR roles in lung fibrosis. **A**–**C** EGFR role in lung injury: **A** Lung injury induces an upregulation of EGFR ligands such as EGF and AREG, that promote EGFR activation signalling leading to airway remodelling. Specifically, EGF-EGFR binding increases squamous metaplasia of basal cells and alters barrier integrity, due to suppression of tight junction. Moreover, EGF promotes EGFR degradation. AREG-EGFR binding induces hyperplasia of basal cells and mucous cell hyperplasia and stimulates expression of genes related to mucous differentiation. Furthermore, AREG leads to EGFR prolonged signal pathways due to EGFR recycling process on the plasma membrane. **B** Oxidative stress activates EGFR-PI3K-AKT signaling pathways, such as FOXO3A, that inhibits NF-κB, leading to the expression of several cytokines that favors lung inflammation. Moreover, AKT activation regulates cell survival. Altogether these events contribute to lung fibrosis development. **C** Oxidative stress induces the formation of IL-33-RACE-EGFR complex that favors the mucin secretion and globet cell differentiation in lung epithelium. **D**, **E** EGFR activation: **D** EGFR transactivation by TNF-α induces activation of caspase8/3-mediated apoptosis, leading to cell death. However, AREG-EGFR binding triggers EGFR phosphorylation, activating AKT signaling that inhibits apoptosis processes. **E** TGF-β increases the expression of AREG on lung fibroblasts via ADAM17, which promotes the ligand release. AREG-EGFR binding induces ERK, JNK and AP‐1 expression, promoting the expression of profibrotic and mesenchymal genes, such as α-smooth muscle actin, collagen 1-α1/α2, fibronectin and tenascin, involved in the ECM remodeling and in the EMT process

Besides controlling lung embryogenesis and morphogenesis, EGFR plays an essential function in lung homeostasis and airway remodeling that is reflected in different forms of chronic obstructive pulmonary disease (COPD), including acute asthma and fibrosis [232–235]. The EGFR ligands EGF and AREG are upregulated after lung injury, such as caused by cigarette smoking, which leads to EGFR signaling induction. The airway remodeling is the principal step that characterizes the lung epithelium exposed to cigarette smoke, resulting in being the primary feature of COPD due to the dysregulation of EGFR signaling. Indeed, cigarette smoke induces an upregulation of EGF in ciliated differentiated cells that stimulates at least in part the AREG expression in basal cells and squamous-like cells. Both ligands bind EGFR in the basal cells where it is usually expressed. They show different effects on airway remodeling over time depending on their affinity for EGFR, resulting in a different fate of receptors. Firstly, the upregulation of EGF promotes a reduction in the number of secretory and ciliated cells, an increase of squamous metaplasia of basal cells, which undergo molecular changes related to the EMT phenotype, and alteration of barrier integrity due to suppression of tight junction. Later, AREG expression via EGFR stimulation, besides suppressing ciliated cell differentiation and barrier integrity as EGF, induces hyperplasia of basal cells and mucous cell hyperplasia, increasing expression of genes related to mucous differentiation, including mucins MUC5AC and MUC5B. AREG negatively modulates EGF expression, leading to an airway phenotype characterized by basal cells and mucous hyperplasia and a decrease in squamous metaplasia (Fig. 13A) [236–238]. These data suggest that the interplay between EGF and AREG ligands results to be necessary for the regulation of EGFR signaling pathways. On the basis of the ligand, EGFR activates the transcription of several different genes that generate specific cellular phenotype changes, resulting in the alteration of airway epithelium. The high expression of genes related to oxidative stress upon EGF upregulation could constitute one of the mechanisms through which EGFR regulates airway remodeling [236]. ROS-mediated oxidative stress is induced by different extracellular stimuli and damages and leads to an alteration of redox homeostasis, which is associated with an increase in the expression of chemokines and cytokines and promotion of tumorigenesis. In response to oxidative stress, EGFR-PI3K-AKT signaling pathways are activated to promote cell survival and suppress cell death via many AKT effectors, including forkhead box transcription factors (FOXOs), c-Myc, hypoxia-inducible factor (HIF), mechanistic target of rapamycin complex 1/2 (mTORC1/2), mTOR substrate S6 kinase 1/2 (S6K1/2), sterol regulatory element-binding protein 1 (SREBP1), and glycogen synthase kinase 3 (GSK3) [239, 240]. During airway lung remodeling, an increase of chemokines and proinflammatory cytokines, such as IL-8, is associated with EGFR phosphorylation and AKT activation. AKT phosphorylates FOXO3A, an inhibitor of NF-κB, decreasing its nuclear expression and increasing its cytoplasmatic accumulation. NF-κB leads to the expression and secretion of several cytokines, that promote lung inflammation by recruitment of inflammatory cells in the lung tissue (Fig. 13B) [241, 242]. Oxidative stress in the lung epithelium induces the upregulation of the oxidized form of Interleukin (IL)−33, which, through expression of its receptor RACE on plasma membrane, forms a complex with EGFR, increasing its activation, in addition to that triggered by EGF or AREG ligands. The IL-33-RACE-EGFR axis regulates the expression of genes involved in mucin secretion and globet cell differentiation, at the expense of ciliated cells (Fig. 13C) [243].

The EGFR-AKT pathway during inflammation of lung epithelium promotes cell survival, contrasting apoptosis. This mechanism of protection may be due to an increase of AREG expression in the alveolar macrophages, together with TNF-α. Generally, TNF-α induces activation of caspase 8/3-mediated apoptosis via transactivation of EGFR, leading to cell death. However, the expression of AREG further enhances EGFR phosphorylation compared to TNF-α, activating AKT signaling that protects cells from apoptosis (Fig. 13D) [244–246].

Persistent respiratory system inflammation leads to bronchial asthma, characterized by airway remodeling, collagen deposition, and fibrosis. Fibrosis involves tissue remodeling, including differentiation of bronchial fibroblasts into myofibroblasts, EMT (which contributes to enhanced synthesis of collagen), and ECM deposition [247, 248]. It is induced mainly by transforming growth factor-β (TGF-β), which plays this function through EGFR pathways. TGF-β increases AREG expression on lung fibroblasts by the action of ADAM17, that promotes the ligand release. Thus, AREG binds and activates EGFR, resulting in the regulation of downstream pathways, such as ERK, JNK and AP‐1, which mediate the expression of profibrotic and mesenchymal genes, such as α-smooth muscle actin, collagen 1-α1/α2, fibronectin and tenascin, involved in the extracellular matrix deposition and in the EMT process (Fig. 13E) [249–251].

These data demonstrate the multiple functions of EGFR and its downstream signaling pathways in lung morphogenesis and tissue homeostasis, which are characterized by a fine regulation of EGFR, resulting in controlled cellular proliferation, differentiation, migration, apoptosis, and survival. As a result, EGFR represents one of the most critical factors in lung development. At the same time, altered expression of its ligands by extracellular stimuli and injury, such as cigarette smoking and consequent oxidative stress, can lead to upregulation of EGFR and activation of its signaling pathways, causing structural and functional alterations in the lung epithelium and impairment of cell phenotype, that contribute to different forms of chronic obstructive pulmonary disease (COPD).

## Conclusions

This review focuses on the expression of the EGFR and its role in regulating normal development of many organs. EGFR interacts with several growth factors and proteins, activating multiple signaling pathways within cells. This allows it to influence many cellular processes, both in healthy tissues and in disease. EGFR activation is essential for controlling cell proliferation, differentiation, and migration. It plays a critical role in maintaining tissue homeostasis, cell function, and the remodeling of the extracellular matrix (ECM). Activators and inhibitors tightly regulate EGFR activity, which is essential for mammalian development.

EGFR promotes the regeneration and repair of injured tissues in various organs by stimulating cell proliferation. However, uncontrolled EGFR activation can be detrimental, leading to fibrosis, inflammation, and even cancer development.

EGFR also plays a vital role in branching morphogenesis, a process crucial for proper organ formation. Altered EGFR expression is associated with developmental defects in various organs.

This review aims to provide an overview of EGFR's role in the development of many organs. By highlighting these diverse functions, we suggest potential therapeutic strategies targeting EGFR to promote healthy development and tissue homeostasis.

## Data Availability

Not applicable.

## Abbreviations

αSyn: α-Synuclein
AD: Alzheimer’s disease
ADAMs: Disintegrin and metalloproteinase
APCs: Astrocyte progenitor cells
APP: Amyloid precursor protein
AREG: Amphiregulin
BAs: Bile acids
BMP: Bone morphogenetic protein
BPS: Bisphenol S
BTC: Betacellulin
CAC: Colitis-associated colorectal
CAMKII: Calcium/Calmodulin Dependent Protein Kinase II
Cdc42: Cell division control 42
CE: Cornified envelope
CKD: Chronic kidney disease
CME: Clathrin-mediated endocytosis
COPD: Chronic obstructive pulmonary disease
cPKC: Protein kinase C
CRC: Colorectal cancer
DcR3: Decoy receptor 3
DDS: Dextran sulfate sodium
ECM: Extracellular matrix
EGF: Epidermal growth factor
EGFR: Epidermal growth factor receptors
EGFRIs: Epidermal growth factor inhibitors
EMT: Epithelial–mesenchymal transition
EPGN: Epigen
ER: Endoplasmic reticulum
EREG: Epiregulin
EVTs: Extravillous trophoblasts
FGFRs: Fibroblast growth factor receptors
FOXOs: Forkhead box transcription factors
GBM: Glioblastoma
GM-CSF: Granulocyte/macrophage-colony stimulating factor
GPCR: G protein-coupled receptor
HB-EGF: Heparin-binding EGF-like growth factor
HCC: Hepatocellular carcinoma
HD: Huntington’s disease
IBD: Inflammatory bowel diseases
ID3: Inhibitor of DNA-binding protein
IGF-1R: Insulin-like growth factor receptors
ISCs: Intestinal stem cells
KA: Kainic acid
KD: Knock-down
KO: Knock-out
LAP: Lapatinib ditosylate
LNPs: Lipid nanoparticles
MANCs: Mesenchymal alveolar niche cells
MET: Mesenchymal–epithelial transition
MHC: Major histocompatibility complex
mHtt: Mutated huntingtin
MMP: Matrix metalloproteinases
MTLE-HS: Mesial Temporal Lobe Epilepsy with Hippocampal Sclerosis
mtTFB2: Dimethyladenosine transferase 2, mitochondrial
NAFLD: Non-alcoholic fatty liver disease
NCE: Non-Clathrin-mediated endocytosis
NICD: Notch1 intracellular domain
NPC: Neuroblast progenitor cells
OA: Osteoarthritis
OPCs: Oligodendrocyte progenitor cells
PD: Parkinson’s disease
PDGFRs: Platelet-derived growth factor receptors
PE: Preeclampsia
PH: Partial hepatectomy
PM: Plasma membrane
PS: Presenilins
React-NSCs: Reactive neural stem cells
RG: Radial glial
RTKs: Tyrosine kinase receptors
RTN3: (ER)-resident protein reticulon-3
sFlt-1: Soluble FMS-like tyrosine kinase-1
SHH: Sonic hedgehog
SNC: Nervous system central
SVZ: Subventricular zone
TCR: T cell receptor
TFAM: Mitochondrial transcription factor A
TGF-α: Transforming growth factor-alpha
TGase: Transglutaminase
TGF-β: Transforming growth factor‐beta
TKI: Tyrosine kinase inhibitors
TME: Tumor microenvironment
Treg: T regulatory cells
UPR: Unfolded protein response
VEGFRs: Vascular endothelial growth factor receptors
